# Ironomycin induces mantle cell lymphoma cell death by targeting iron metabolism addiction

**DOI:** 10.7150/thno.101821

**Published:** 2025-02-03

**Authors:** Sara Ovejero, Laura Alibert, Julie Devin, Tatiana Cañeque, Valentin Jacquier, Andrea Romero, Salome Amar, Matthieu Abouladze, Elvira Garcia de Paco, Ouissem Karmous Gadacha, Guilhem Requirand, Nicolas Robert, Miss Leriem Zellagui, Hugues de Boussac, Guillaume Cartron, Johanna Chiche, Jean-Ehrland Ricci, Charles Herbaux, Raphael Rodriguez, Jerome Moreaux, Caroline Bret

**Affiliations:** 1 Institute of Human Genetics UMR 9002 CNRS-UM, Montpellier, France.; 2 Department of Biological Hematology, CHU Montpellier, Montpellier, France.; 3 Chemical Biology of Cancer Laboratory, Institut Curie, 26 rue d'Ulm, 75248 Paris Cedex 05, France; PSL Université, Paris, France; CNRS UMR 3666, Paris, France; INSERM U1143, Paris, France.; 4 Diag2Tec, Montpellier, France.; 5 Department of Clinical Hematology, CHU Montpellier, Montpellier, France.; 6 CNRS UMR 5535, University of Montpellier, Montpellier, France.; 7 Institut Universitaire de France, Paris, France.; 8 Université Côte d'Azur, INSERM, C3M, Nice, France.; 9 Équipe labellisée LIGUE Contre le Cancer, Nice, France.

**Keywords:** iron metabolism, mantle cell lymphoma, ironomycin, drug combination, B-cell receptor signaling

## Abstract

**Rationale:** Mantle-cell lymphoma (MCL) remains an aggressive and incurable cancer. Accumulating evidence reveals that abnormal iron metabolism plays an important role in tumorigenesis and in cancer progression of many tumors. Based on these data, we searched to identify alterations of iron homeostasis in MCL that could be exploited to develop novel therapeutic strategies.

**Methods:** Analysis of the iron metabolism gene expression profile of a cohort of patients with MCL enables the identification of patients with a poor outcome who might benefit from an iron homeostasis-targeted therapy. We analyzed the therapeutic interest of ironomycin, known to sequester iron in the lysosome and to induce ferroptosis.

**Results:** In a panel of MCL cell lines, ironomycin inhibited MCL cell growth at nanomolar concentrations compared with conventional iron chelators. Ironomycin treatment resulted in ferroptosis induction and decreased cell proliferation rate, with a reduced percentage of cells in S-phase together with Ki67 and Cyclin D1 downregulation. Ironomycin treatment induced DNA damage response, accumulation of DNA double-strand breaks, and activated the Unfolded Protein Response (UPR). We validated the therapeutic interest of ironomycin in primary MCL cells of patients. Ironomycin demonstrated a significant higher toxicity in MCL cells compared to normal cells from the microenvironment. We tested the therapeutic interest of combining ironomycin with conventional treatments used in MCL. We identified a synergistic effect when ironomycin is combined with Ibrutinib, Bruton's tyrosine kinase (BTK) inhibitor, associated with a strong inhibition of B-Cell receptor (BCR) signaling.

**Conclusion:** Altogether, these data underline that MCL patients my benefit from targeting iron homeostasis using ironomycin alone or in combination with conventional MCL treatments.

## Introduction

Mantle cell lymphoma (MCL) is a rare subtype of non-Hodgkin's lymphoma (NHL), that accounts for 5-7% of all NHL cases. MCL is derived from mostly antigen-naïve cells that proliferate in the mantle zone around germinal centers. One of the main genetic characteristics of MCL is chromosome translocation t(11;14) that causes Cyclin D1 overexpression, conferring a proliferative phenotype to tumor cells 1. In addition, aberrations of *TP53* in aggressive MCL have a negative impact on survival 2.

The median age of MCL patients is 60-70 years 1. Despite recent advances, it remains incurable and patients with high-risk disease have particularly poor outcomes. Depending on the age and fitness of the patient, treatments include conventional chemotherapy and stem cell transplantation (SCT), BTK inhibitors, or bispecific antibodies against CD19 and CD20, among others. However, drug resistance and disease progression are major challenges in the treatment of MCL 2.

Ibrutinib inhibits BTK, thereby blocking BCR signaling, which is abnormally active in some B-cell cancers, including lymphomas. Ibrutinib is approved to treat MCL patients that have received at least one previous line of treatment 3. In addition, oral BTK inhibitors administered alone 4, or combinations of ibrutinib with rituximab 5, or ibrutinib with the Bcl2-antagonist venetoclax 6,7, have proven as interesting chemotherapy-free targeted therapeutic approaches for MCL patients at relapse 8. However, primary and acquired resistance to ibrutinib has already been described in MCL patients 9. Thus, the study of the mechanisms of cancer cell resistance to ibrutinib and response to its combination with other drugs is of great therapeutic interest in treating patients with MCL.

Iron is an essential element for cells. It is a critical component of many biological processes such as mitochondrial function 10, DNA replication and repair 11, and epigenetic modifications 12. Iron is also a redox-active metal that can participate in free radical formation and propagation of lipid peroxidation through the Fenton reaction, which can cause a type of iron-dependent non-apoptotic cell death known as ferroptosis 13. Thus, iron dysregulation is linked to pathological states 14. Indeed, cancer cells often present dysregulation of many genes involved in iron metabolism, and abnormal iron homeostasis is involved in autoimmunity, tumorigenesis, and the progression of cancers 15,16. In the past years, inducing ferroptosis with iron-targeting molecules, such as iron chelators or iron oxide nanoparticles, has gained attention as a promising anti-cancer strategy in hematologic malignancies 17-21.

Considering the importance of iron homeostasis in cell biology and its implication in cancer, we investigated the therapeutic potential of targeting the iron pool of MCL cells with ironomycin, a promising agent known for sequestering iron in lysosomes and inducing cancer cell death 22,23. Our findings demonstrate that ironomycin triggers both apoptosis and ferroptosis in MCL cells. Ironomycin also activates the UPR pathway, a cellular stress response triggered by the accumulation of misfolded or unfolded proteins in the endoplasmic reticulum (ER). Moreover, we observed a synergistic effect when ironomycin is combined with ibrutinib, leading to increased MCL cell death, suggesting that there is a therapeutic benefit in the combined approach of BCR inhibition and iron homeostasis targeting for the treatment of MCL patients.

## Methods

### Mantle cell lymphoma cell line culture

6 MCL cell lines (JEKO1, JVM2, MAVER1, MINO, REC1, GRANTA519) were purchased from the DSMZ (Leibniz-Institut DSMZ - GmbH, Germany). They were cultured in RPMI with 10% FBS (JVM2, REC1) or 20% FBS (JEKO1, MAVER1, MINO); or DMEM with 10% FBS (GRANTA519) at 37 °C and 5% CO_2_. Cells were passed every 3-4 days.

### Reagents

Ironomycin (AM5) was a kind gift from Raphaël Rodriguez (patent application WO2016/038223).

Deferasirox (ITM101102264, TargetMol), Erastin (S7242, Selleckchem), Ferrostatin-1 (S7243, Selleckchem), Q-VD Oph (S7311, SelleckChem), Iron (III) Chloride Hexahydrate (31232-M, Sigma Aldrich), N-Acetyl Cysteine (A9165, Sigma Aldrich), Ibrutinib (S2680, Sellekchem), Venetoclax (S8048, Sellekchem), AZD-5991 (S8643, Selleckchem), A1155463 (T6748, TargetMol), bendamustine (S5939, Sellekchem), bortezomib (S1013, Selleckchem).

### Synergy matrices

For evaluation of ironomycin synergy with ibrutinib, venetoclax, AZ1159XX and A-1155463 , cells were seeded at 30000 (JVM2) or 50000 (JEKO1, MAVER1) cells/well and cultured for 4 days in 96-well flat-bottom plates in the presence of ironomycin (0.01 - 4 μM), ibrutinib (BTK inhibitor; 0.125 - 32 μM), venetoclax (Bcl2 inhibitor; JEKO1/JVM2: 125 - 32000 nM; MAVER1: 0.12 - 8000 nM), AZ1159 (Mcl1 inhibitor; 0.01 - 5 μM), A1155463 (Bcl-xL inhibitor: 0.15 - 40 μM). Increasing concentrations of ironomycin were combined with increasing concentrations of the other drugs to evaluate all possible combinations. Cell growth was evaluated using CellTiter-Glo (CTG) Luminiscent Assay (G7573, Promega) according to manufacturer's protocol and luminescence was measured using a Centro LB 960 luminometer (Berthold Technologies). For each combination, the percentage of expected growing cells in the case of effect independence was calculated with Bliss equation using R package “SynergyFinder”.

Supplementary information is included in Supplemental Methods.

## Results

### Iron homeostasis-related gene expression profile identifies high risk MCL patients

**Figure 1:**
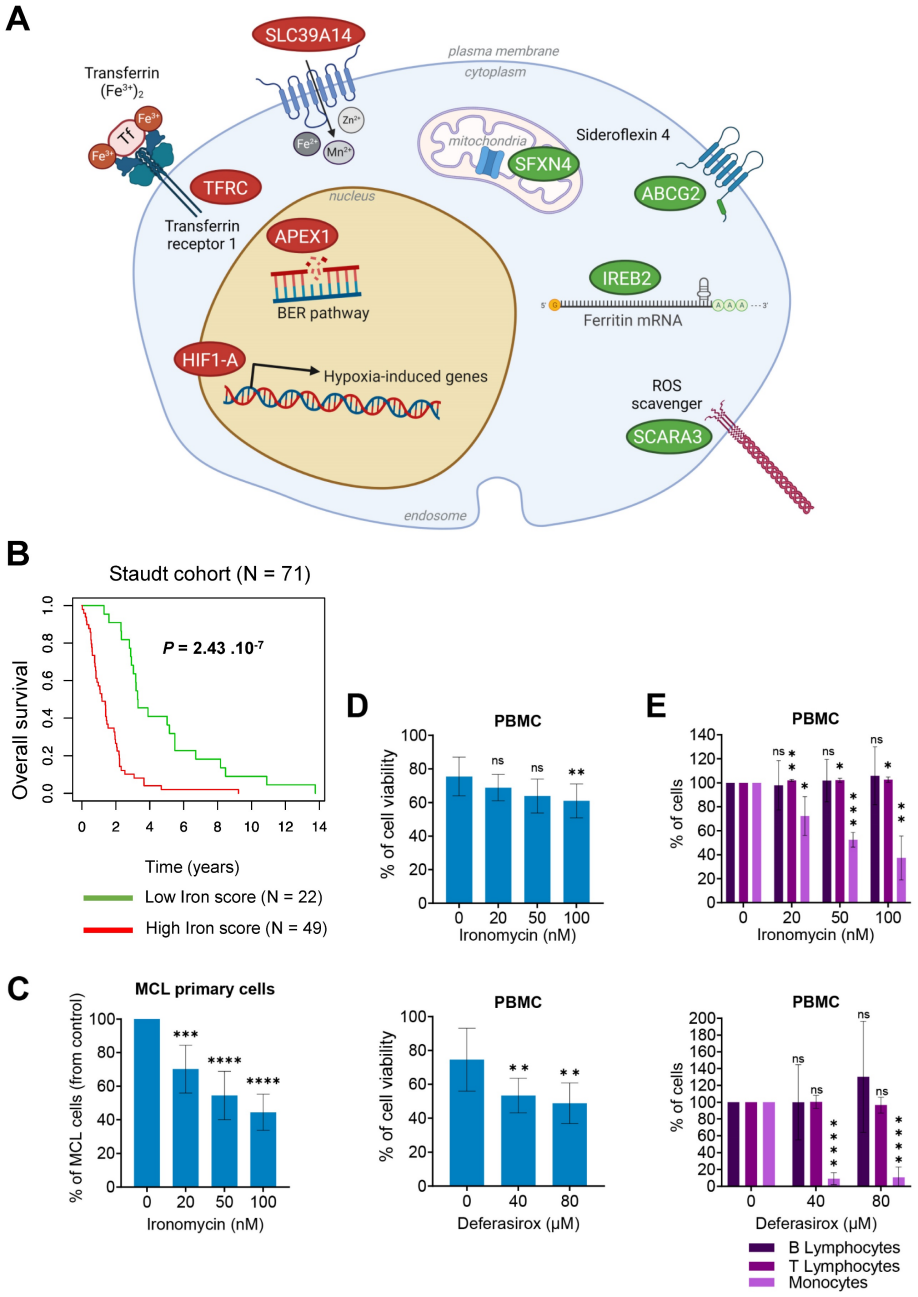
** The iron score predicts the clinical outcome in MCL. (A)** A list of 62 genes involved in the regulation of iron biology was established using previously published data 20,24. Gene expression microarray data from one cohort (Staudt cohort) of 71 newly-diagnosed MCL patients was used (accession number GSE10793). Data were analyzed with Microarray Suite version 5.0 (MAS 5.0), using Affymetrix default analysis settings and global scaling as normalization method. The trimmed mean target intensity of each array was arbitrarily set to 500. 4 iron-related genes were found to have a good prognostic value (in green) and 4 a bad prognostic value (in red). *ABCG2* (ATP-binding cassette transporter G2), *SCARA3* (Scavenger Receptor Class A Member 3), *IREB2* (Iron Responsive Element Binding Protein 2) and *SFXN4* (sideroflexin 4); (*APEX1* (DNA-(apurinic or apyrimidinic site) lyase), *TFRC* (Transferrin Receptor Protein 1), *SLC39A14* (Solute Carrier Family 39 Member 14), and *HIF1A* (Hypoxia inducible factor A 1). Scheme was created with BioRender.** (B)** Patients of the Staudt cohort GSE10793 (n = 71) were ranked according to increased iron score and a maximum difference in OS (overall survival) was obtained with iron score of -3.7798 (also named 'cut point') splitting patients into high-risk and low-risk groups. The iron score was significantly associated with high-risk in MCL patients. **(C)** Primary MCL cells from 9 patients were treated with ironomycin at the indicated concentrations for 4 days. Tumor cells were analyzed by flow cytometry and expressed in % of control. Results represent the median ± IQR. Statistical significance was tested using paired t-test: *** p value < 0.001, **** p value < 0.0001. **(D,E)** Peripheral blood mononucleated cells (PBMC) from healthy donors (n = 5) were treated with ironomycin or deferasirox for 4 days, counted in presence of trypan blue to visually distinguish dead cells (trypan blue positive) from living cells (trypan blue negative). (D) Viability was calculated as the percentage of living cells to total cells (living + dead) in each condition compared to control. (E) Populations of B-lymphocytes, T-lymphocytes and monocytes were quantified by flow cytometry and compared to control condition. The 3 populations are expressed as % of control. Asterisks indicate significant differences compared to control conditions after applying a Student's t-test for pairs. *: p-value < 0.05; **: p-value < 0.01; ***: p: value < 0.001; ns: not significant.

According to the major role of iron homeostasis in cancer, we aimed to identify iron metabolism-related genes associated with a prognostic value in MCL. Sixty-two genes related to iron biology and cancer had been reported 20,24 (Table S1). Using Maxstat R algorithm 25 and multiple testing correction, we identified 8 genes with significant prognostic value in a cohort of newly diagnosed MCL patients (n = 71) 26 (Figure 1A) and combined their prognostic information in a Gene Expression Profile (GEP)-based iron-score (IS) as previously described 27,28. IS is defined by the sum of the beta coefficients of the Cox model for each prognostic gene, weighted by +1 or -1 according to the patient expression signal above or below the Maxstat value. IS segregated the cohort in two groups (iron-score cut point: -3.7798) with a maximum difference in overall survival (OS; Figure 1B), underlining that an elevated IS allows the identification of MCL patients with poor prognosis and dysregulation of iron metabolism who could benefit from targeted therapy.

### Targeting iron homeostasis kills MCL cells

We and others reported the therapeutic interest of targeting iron homeostasis with ironomycin to kill Diffuse Large B-Cell Lymphoma (DLBCL) 20 and Acute Myeloid Leukemia (AML) 29 cells. Using 6 MCL cell lines, we determined the IC50 of ironomycin. Deferasirox, an iron chelator 30-32 approved by the FDA to treat chronic iron overload by selectively binding the ferric form of iron 33, was used as a control. Of note, deferasirox was evaluated in MCL cells 19 and reported to have anti-tumoral effects *in vitro* 34. Interestingly, IC50 values of ironomycin were in the nanomolar range, whereas those of deferasirox were in the micromolar range (Figure S1A), indicating that ironomycin is ~100-fold more potent in inhibiting MCL cells growth. Ironomycin is also significantly toxic to MCL primary cells at nanomolar concentrations (Figure 1C and Figure S1B). Furthermore, only deferasirox significantly impacted viability of purified peripheral blood mononucleated cells (PBMC) from healthy donors (Figure 1D). Both ironomycin and deferasirox were not toxic to normal B- and T-lymphocytes, but killed monocytes in a dose-dependent manner (Figure 1E). Monocytes are known to participate in iron recycling and accumulate intracellular iron 35 which makes them more susceptible to drugs targeting iron homeostasis. Moreover, a small but significant increase in the percentage of T-lymphocytes was also observed exclusively upon ironomycin treatment.

**Figure 2:**
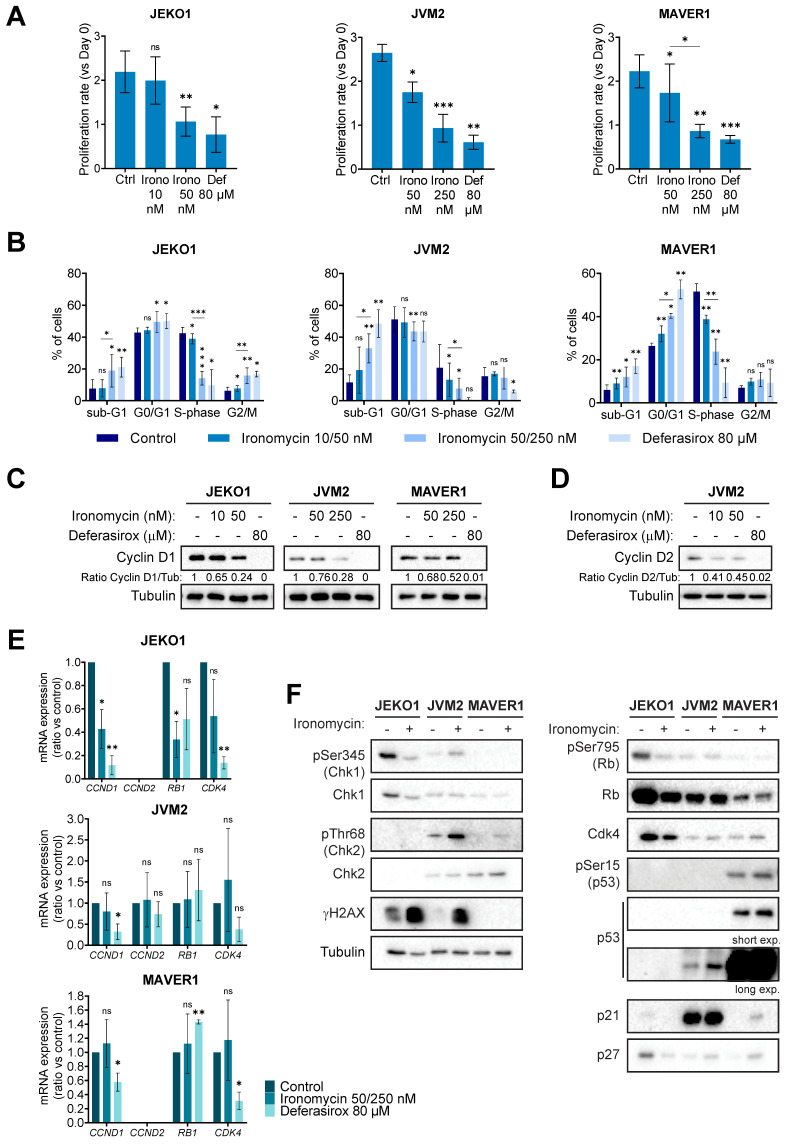
** Ironomycin impairs the proliferation of MCL cells. (A)** JEKO1, JVM2 and MAVER1 cell lines were treated as indicated for 48 h. Cells were counted at day 0 and at the end of the treatments, and the number of cells was normalized to day 0 to calculate the proliferation rate. Graphs show the average and standard deviation of 3-4 independent experiments. **(B)** Cells were treated or not with ironomycin (JEKO1: 10 and 50 nM; JVM2/MAVER1: 50 and 250 nM) and Deferasirox (80 µM) for 48 h and BrdU (10 μg/ml) was added during the last 1.5 h of treatment. Cells were fixed and processed to detect BrdU incorporation and total DNA. BrdU+ cells were assigned to S-phase. BrdU- cells were assigned to G0/G1 or G2/M phases based on their DNA content. Results are the mean of 3-4 independent experiments. **(C,D)** Cells were treated as indicated for 48 h, and the levels of Cyclin D1 and Cyclin D2 were analyze in cell lysates by western blot. Tubulin was used as a loading control. Figures show 1 representative experiment out of 3. **(E)** Total mRNA was extracted from cells treated as indicated for 48 h, subjected to retrotranscription and the levels of expression of *CCND1*, *CCND2*, *RB1* and *CDK4* genes were quantified by qPCR. Graphs show the average ± SD of 3 independent experiments. **(F)** Cells were treated or not with ironomycin (JEKO1: 50 nM; JVM2/MAVER1: 250 nM) for 48 h, collected and the indicated proteins were analyzed by western blot in whole cell lysates. In all the graphs in this figure, asterisks indicate significant differences compared to control conditions after applying a Student's t-test for pairs. *: p-value < 0.05; **: p-value < 0.01; ***: p-value < 0.001; ns: not significant.

Then, to further characterize the biological effect of ironomycin on MCL cells, we chose 3 cell lines (JEKO1, JVM2 and MAVER1) with different ironomycin IC50 and that partially represent the molecular heterogeneity of MCL patients (Table S2). High concentration of deferasirox was used to compare the effect of iron chelation versus ironomycin-induced iron sequestration. Ironomycin treatment induced a decrease in proliferation (Figure 2A and Figure S1C) associated with an altered cell cycle distribution (Figure 2B). In MCL, t(11;14) translocation, which causes the over-expression of the gene *CCND1,* is associated with high tumor cell proliferation 36. JVM2 expresses lower protein levels of Cyclin D1 than other MCL cell lines, while co-expressing Cyclin D2 37 (Table S2). Importantly, treatment of MCL cells with ironomycin caused a marked diminution of Cyclin D1 and Cyclin D2 protein levels (Figure 2C,D). We confirmed that the decrease in Cyclin D1 expression correlated with a decrease in *CCND1* transcription in JEKO1. However, no difference in mRNA levels of *CCND1/CCND2* were observed in JVM2/MAVER1 (Figure 2E), suggesting that lower protein levels may be due to increased protein degradation. Cyclin D1 is degraded by the proteasome and it was reported that deferasirox induces proteasome-mediated Cyclin D1 degradation 19,34. Indeed, proteasome inhibitor bortezomib rescued Cyclin D1/Cyclin D2 degradation induced by ironomycin and deferasirox (Figure S1D).

Furthermore, we analyzed whether ironomycin also impacted the levels of several well-known factors controlling cell cycle progression and linked to Cyclin D1 (Cdk4, Rb, p53, p21 and p27) 36. It has been reported that these MCL cell lines present different abnormalities regarding some of these genes (Table S2) and we confirmed different protein levels by western blot (Figure 2F). Interestingly, in JEKO1 and JVM2, ironomycin induced γH2AX, a marker of DNA double strand breaks and DNA damage response (DDR) activation 38. We did not observe γH2AX in MAVER1, probably due to the inactivation of ATM in this cell line (Table S2), but Chk2 was slightly phosphorylated in response to ironomycin.

### Ironomycin causes cell death mediated by apoptosis in MCL cells

**Figure 3:**
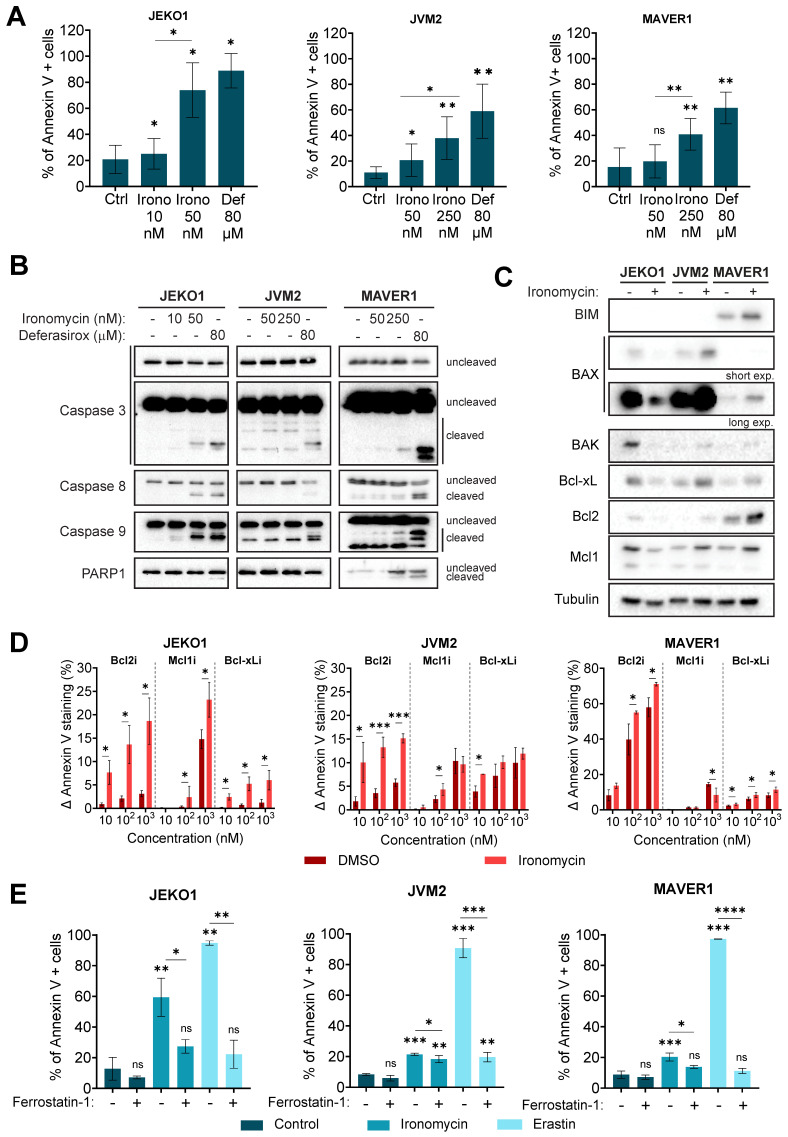
** (A)** Cells were treated as indicated for 48 h and Annexin V was detected by flow cytometry. Results are the mean ± SD of 3 independent experiments. **(B)** Cells were treated as in (A). The levels of the indicated proteins were analyzed by western blot. Figure shows 1 representative out of 3 independent experiments. **(C)** Cells were treated with ironomycin (JEKO1: 50 nM, JVM2/MAVER1: 250 nM) for 48 h, and the levels of the indicated proteins were analyzed by western blot. Tubulin was used as a loading control. Figure shows 1 representative out of 3 independent experiments. **(D)** BH3 profiling of JEKO1, JVM2 and MAVER1. Cells were treated with ironomycin (JEKO1: 50 nM, JVM2/MAVER1: 250 nM) or DMSO for 20 h. Then, BH3 mimetics (venetoclax: Bcl2i, AZD-5991: Mcl1i, A-1155463: Bcl-xLi) or vehicle DMSO (control) were added to the culture medium for 4 h. Annexin V+ cells were detected by flow cytometry. Graphs represent the difference (Δ) between the percentage of Annexin V+ cells in each condition and in the control (vehicle DMSO). Results are the mean ± SD of 3 independent experiments. **(E)** Cells were pre-treated with the ferroptosis inhibitor Ferrostatin-1 (10 μM, 30 min) before treatment with ironomycin (JEKO1: 50 nM; JVM2/MAVER1: 250 nM) or the ferroptosis inducer erastin (4 μM) for 48 h. Annexin V was detected by flow cytometry. Graphs show the mean ± SD of 3-4 independent experiments. In all the graphs in this figure, asterisks indicate significant differences compared to control conditions after applying a Student's t-test for pairs. *: p-value < 0.05; **: p-value < 0.01; ***: p-value < 0.001; ****: p-value < 0.0001; ns: not significant.

Ironomycin significantly reduced cell viability in all cell lines (Figure S1E). Since ironomycin has been reported to induce ferroptosis, apoptosis and ferritinophagy 20,22,23,29, we sought to identify the type(s) of cell death that it induces in MCL cells. Ironomycin and deferasirox increased the Annexin V+ population (Figure 3A), indicative of apoptotic cell death. Supplementation of cells with FeCl_3_ prevented cell death caused by deferasirox, but not by ironomycin (Figure S2A).

Increase in Annexin V+ cells upon ironomycin treatment correlated with caspases 3, 8 and 9 cleavage in JEKO1 and MAVER1 cell lines, but not in JVM2 (Figure 3B). Given the limited sensitivity of western blot analysis and the fact that it has been described that caspases 8 and 9 activity is stimulated by dimerization instead of cleavage 39, we confirmed activation of caspases by the more sensitive CaspaseGlo® Assay. We observed that ironomycin increased caspase 3 and 8 activity in all three cell lines, although it was only statistically significant in JEKO1 and JVM2, whereas caspase 9 was only significantly activated in JEKO1 (Figure S2B). Interestingly, pre-treatment of cells with pancaspase inhibitor Quinoline-Val-Asp-Difluorophenoxymethylketone (Q-VD-Oph) partially rescued cell death in JEKO1 and JVM2 cell lines, suggesting that apoptosis is not the only cell death type induced by ironomycin in MCL cells (Figure S2C).

Cancer cells are frequently addicted to the presence of anti-apoptotic factors, such as the Bcl2 family of proteins, that are attractive therapeutic targets 40. We observed significant changes in the levels of Bcl family anti- and pro-apoptotic factors upon ironomycin treatment that were cell line dependent. In JEKO1 cells, ironomycin induced the degradation of all factors, whereas it caused an increase in JVM2 and MAVER1 (Figure 3C). Given these differences, we used the complementary *in vitro* assay BH3 profiling 41 to measure the apoptotic priming of cells and their dependences on the anti-apoptotic proteins Bcl2, Bcl-xL and Mcl1 upon drug treatment (Figure S3A). We observed an increased dependence on these proteins in JEKO1, and specially to Bcl2 in JVM2/MAVER1 (Figure 3D). Moreover, combining ironomycin with Bcl2i, Bcl-xLi and Mcl1i, resulted in synergistic effects across all cell lines, confirming that ironomycin sensitizes MCL cells to Bcl2-family inhibitors (Figure S3B-D) that have been evaluated in relapsed MCL patients with promising results 42,43.

In response to ironomycin, Cytochrome C level was decreased in JEKO1, and increased in JVM2/MAVER1 (Figure S4A). It was reported that up-regulation of Cytochrome C is linked to caspase activation and triggering of cell death 44. In addition, severe mitochondria damage is associated with higher Cytochrome C release into the extracellular space and higher cell death levels 45. Therefore, the different levels of Cytochrome C in the cell lines may be explained by their different sensitivity to ironomycin. Thus, it is possible that Cytochrome C increase in JVM2 and MAVER1 is linked to moderate apoptosis level and its decrease in JEKO1 may be due to higher levels of cell death (Figure 3A) and loss of cell membrane integrity, which will release cytosolic proteins like Cytochrome C to the medium, that will then be less abundant by western blot. To test this hypothesis, we removed JEKO1 dead cells by Ficoll® centrifugation and performed western blot only on living cells with unbroken cell membrane. The levels of Cytochrome C did not decrease in these cells in ironomycin vs untreated conditions (Figure S4B). In contrast to our previous result (Figure 3C), after dead cell removal, the only anti-apoptotic factor that was actually decreased by ironomycin in JEKO1 cells was Mcl1, whereas the pro-apoptotic proteins Bax and Bak remained unchanged (Figure S4B). In basal conditions, Cytochrome C is necessary for ATP production in the mitochondria and needs iron. We treated cells with increasing doses of ironomycin for 48 h and used CellTiter Glo assay to quantify intracellular ATP. Our results showed a significant dose-dependent decrease in the levels of ATP (Figure S4C), most likely due to iron depletion caused by ironomycin. Using Seahorse functional assay, we confirmed that ironomycin strongly decreased both basal and maximal mitochondrial respiration capacities (Figure S4D). These data indicate that ironomycin impairs mitochondrial function, eventually triggering caspase-dependent apoptosis.

### Ironomycin induces ferroptosis in MCL cells

Ferrostatin-1 46, a ferroptosis inhibitor, rescued ironomycin- and erastin-induced cell death (Figure 3E), confirming that ironomycin also induces ferroptosis in MCL cells. Erastin was used as a positive control 47. Finally, we studied if ironomycin activated autophagy in MCL cells. BIX1294 was used as a positive control 48. No formation of LC3B foci 49 was observed upon ironomycin treatment (Figure S4E). However, western blot analysis showed a modest increase in LC3B-II in JVM2, and a degradation (JEKO1 and MAVER1) or accumulation (JVM2) of ferritin, an iron-storage protein which is degraded when ferritinophagy is activated 50 (Figure S4F,G). Since ferritinophagy triggers ferroptosis and our western blot analysis showed differences in Ferritin and LC3B levels between the three cell lines (Figure S4G), we cannot exclude that ferritinophagy also contributes to ferroptosis initiation in MCL cells. Interestingly, increased expression of *TFRC*, which codes for the transferrin receptor CD71 and has a prognostic value according to our analysis (Figure 1A,B), is associated with more aggressive forms and poor prognosis in MCL 51. We confirmed higher levels of CD71 in MCL cell lines and primary MCL cells from patients than in PBMC from healthy donors (Figure S4H). Furthermore, upon treatment with ironomycin, we observed an increase in CD71 protein levels, a marker of ferroptosis (Figure S4I). Finally, we pretreated cells with Q-VD-Oph, ferrostatin-1 or combination of both (Figure S4J), and confirmed that ironomycin triggers both apoptosis and ferroptosis in MCL cells.

Ironomycin has been involved in the generation of ROS, that cause lipid peroxidation and DNA damage 20 (Figure S4F). In agreement, we observed a small but significant increase in ROS production induced by ironomycin that could not be rescued by iron supplementation (Figure S5A). Intriguingly, combination of exogenous iron and ironomycin led to increased ROS production in JEKO1 and JVM2 compared to ironomycin alone, while it reverted ROS production in combination with deferasirox in JVM2 and MAVER1. Of note, JEKO1 showed an elevated level of ROS already in basal conditions (Figure S5B), that correlated with γH2AX indicative of DNA damage (Figure 2F). This elevated basal ROS level may contribute to the stronger sensitivity of JEKO1 to ironomycin treatment compared to JVM2, in which a 5 times higher concentration of ironomycin was required to reach similar levels of γH2AX (Figure 2F). Given the central role of iron in mitochondria, we also analyzed the production of mitochondrial ROS using the specific probe MitoSox. No significant increase of mitochondrial ROS was detected upon ironomycin or deferasirox treatment (Figure S5C). Given that ROS cause lipid peroxidation that in turn triggers ferroptosis, we used BODIPY dye to monitor lipid oxidation state. BODIPY underlined a significant increase in peroxidized lipids upon ironomycin and erastin treatments, that was diminished by ferrostatin-1 (Figure S5D). The phospholipid hydroperoxidase GPX4 protects cells against membrane lipid peroxidation and is involved in ferroptosis regulation 50. Intriguingly, GPX4 levels varied differently in each MCL cell line in response to ironomycin (Figure S4A,B).

Vitamin E is an antioxidant that has been reported to prevent ferroptosis 52. High-density lipoproteins (HDL) and low-density lipoproteins (LDL) can carry Vitamin E to cells to mitigate lipid peroxidation and ferroptosis 53. In order to evaluate the contribution of lipids to the cellular effects of ironomycin, we cultured cells in medium supplemented with lipid-free serum. Lack of exogenous lipids induced cell death in untreated JVM2 and MAVER1 cell lines, with no effect on JEKO1, suggesting that the three cell lines are metabolically different in basal conditions (Figure S6A). Treatment with ironomycin in absence of lipids only increased cell death in JVM2 cells. ROS production was diminished in JEKO1 and MAVER1 in lipid-depleted medium, but not in JVM2 (Figure S6B). Lipid peroxidation was increased in absence of lipids in all cell lines, and treatment with ironomycin led to a small but significant increase in JEKO1 and JVM2 (Figure S6C). Scavenger Receptor Class B Type I (SR-B1) is an HDL receptor that facilitates cholesterol esters uptake and the bi-directional flux of free cholesterol. SR-B1 has been reported as a mediator of oxidative events in cancer 54. Western blot analysis showed that the three MCL cell lines presented different levels of expression of SR-B1 that were not changed by ironomycin (Figure S6D). We monitored the presence of lipid droplets, the organelles that store triacylglycerols and sterol esters, using the Nile Red dye, which marks polar and neutral lipids including cholesterol esters 55. Surprisingly, no lipid droplets were observed in JVM2, whereas ironomycin decreased lipid droplets in both JEKO1 and MAVER1 (Figure S6E).

We have shown that iron supplementation was not able to rescue ironomycin-induced cell death (Figure S2A), but we investigated if any of the cellular responses induced by ironomycin treatment could be reversed by iron supplementation. As before, deferasirox was used as a control. Addition of iron rescued the degradation of Cyclin D1, ATF6, Bcl-xL and Mcl1 caused by deferasirox, as well as the increase in γH2AX in the three cell lines. However, no consistent changes in protein abundance were detected upon iron supplementation in ironomycin-treated cells, with JVM2 being the only cell line in which the accumulation of Bcl-xL, Bcl2, GPX4 and Cytochrome C was reverted by FeCl_3_ addition (Figure S7). The numerous cellular effects of ironomycin in the three MCL cell lines studied are summarized in Table S3.

### Ironomycin induces dysregulation of BCR pathway

**Figure 4:**
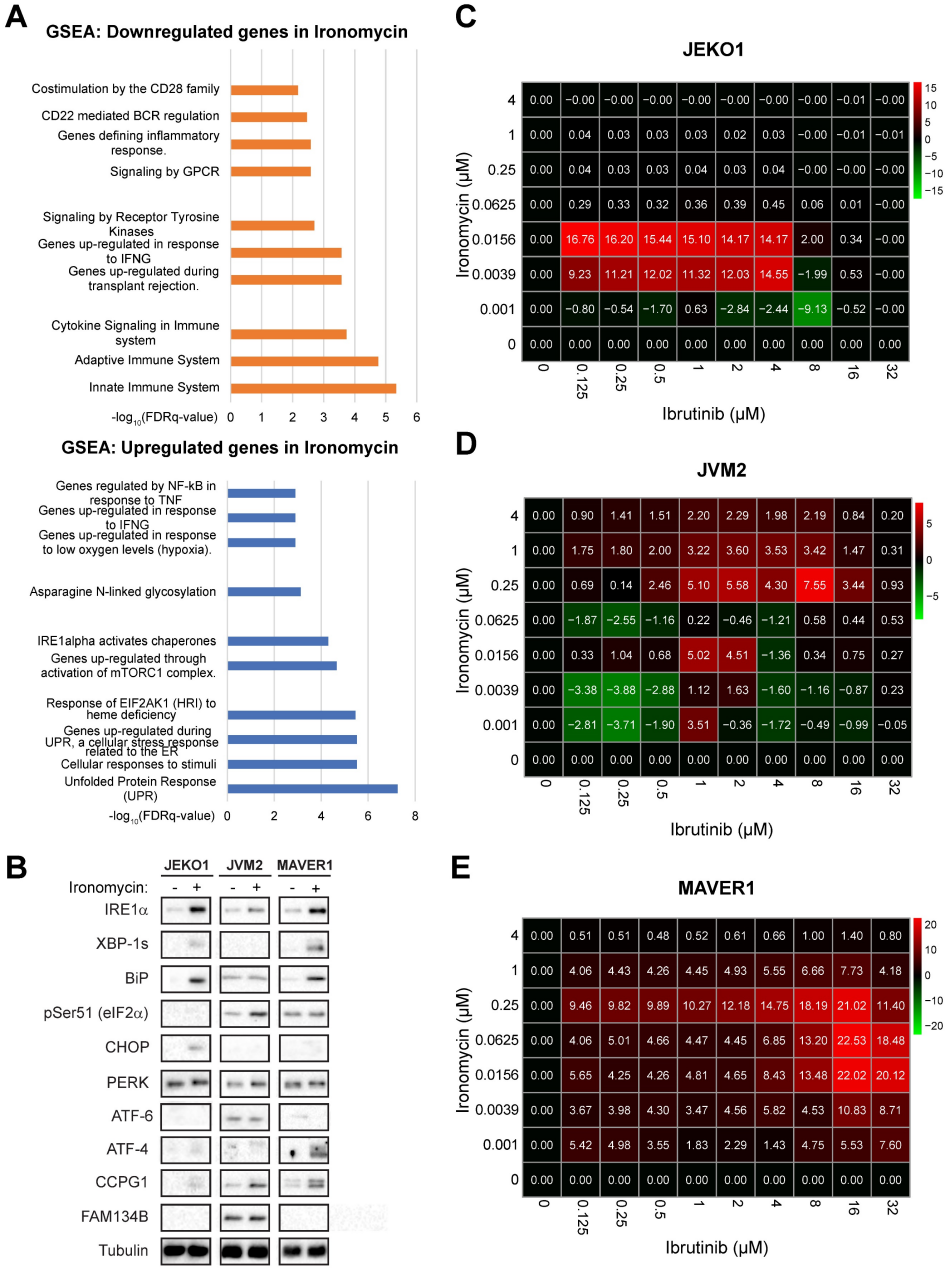
** Ironomycin downregulates the expression of BCR-related genes and synergizes with BTK inhibitor ibrutinib**. **(A)** JEKO1, JVM2 and MAVER1 cells were treated with ironomycin (JEKO1: 50 nM; JVM2/MAVER1: 250 nM) for 48 h. Total RNA was extracted and RNA-sequencing was performed. GSEA of down- and up-regulated pathways is shown. FDR: false discovery rate. **(B)** Cells were treated with ironomycin (JEKO1: 50 nM, JVM2/MAVER1: 250 nM) for 48 h, and the levels of the indicated proteins were analyzed by western blot. Tubulin was used as a loading control. Figure shows 1 representative out of 3 independent experiments. **(C-E)** Cells were seeded in flat-bottom 96-well plates, treated with increasing concentrations of ironomycin (1 - 4000 nM) and ibrutinib (0.125 - 32 μM), and incubated for 4 days. Cell growth was assessed by CellTiter Glo® assay. Drug synergy was calculated using R package “SynergyFinder”. Effect of drug combination on cell growth is shown in a pseudo-color scale from red (synergism) to green (antagonism). Matrixes show the mean of 3 independent experiments.

In order to better understand the global effect of targeting iron homeostasis in MCL, we performed RNA-sequencing (RNA-seq) analysis of MCL cell lines treated with ironomycin. Among the 174 genes significantly differentially expressed, Gene Set Enrichment Analysis (GSEA) showed that UPR was the most upregulated pathway by ironomycin treatment, whereas innate immune system pathways were the most downregulated (Figure 4A). We confirmed that ironomycin induced the accumulation or phosphorylation of several UPR proteins, including IRE1α as well as the generation of XBP1s, indicative of UPR signaling activation (Figure 4B).

Regarding downregulated pathways identified by RNA-seq (Figure 4A), we hypothesized that downregulation of BCR-related genes induced by ironomycin could potentiate the cytotoxic effect of BCR-inhibiting therapy in MCL. Aberrant BCR activation is a key pro-survival pathway that includes BTK, NF-κB and AKT. Ibrutinib is an inhibitor of BTK used in the treatment of MCL. However, drug resistance frequently leads to patient relapse 56. JEKO1 and JVM2 are ibrutinib-sensitive or mild-sensitive cell lines, whereas MAVER1 is resistant (Figure S8A). Using synergy matrixes, we found that ironomycin and ibrutinib synergize to inhibit MCL cells growth (Figure 4C-E). Interestingly, ironomycin combined with ibrutinib induced a downregulation of genes involved in the BCR signaling pathway including *CARD11*, *CD22*, *PTPN6*, *IGLV1-47* and *IGLV1-44* (Figure S8B) 56. We confirmed CARD11 downregulation by western blot (Figure S8C). These results highlight the therapeutic potential of combining ironomycin and ibrutinib to enhance the cytotoxic effects of BTK inhibition, even in ibrutinib-resistant MCL cells.

In order to understand the molecular mechanism of this synergy, we studied if ironomycin could regulate the activation of the BCR pathway. In basal conditions, MCL cell lines presented different activation level of BCR downstream pathways. Drug combination inhibited NF-κB in JEKO1, and BTK and Akt in JVM2. In MAVER1 cell line, the only significant ironomycin effect was the decrease of CARD11 (Figure S8C-E).

**Figure 5:**
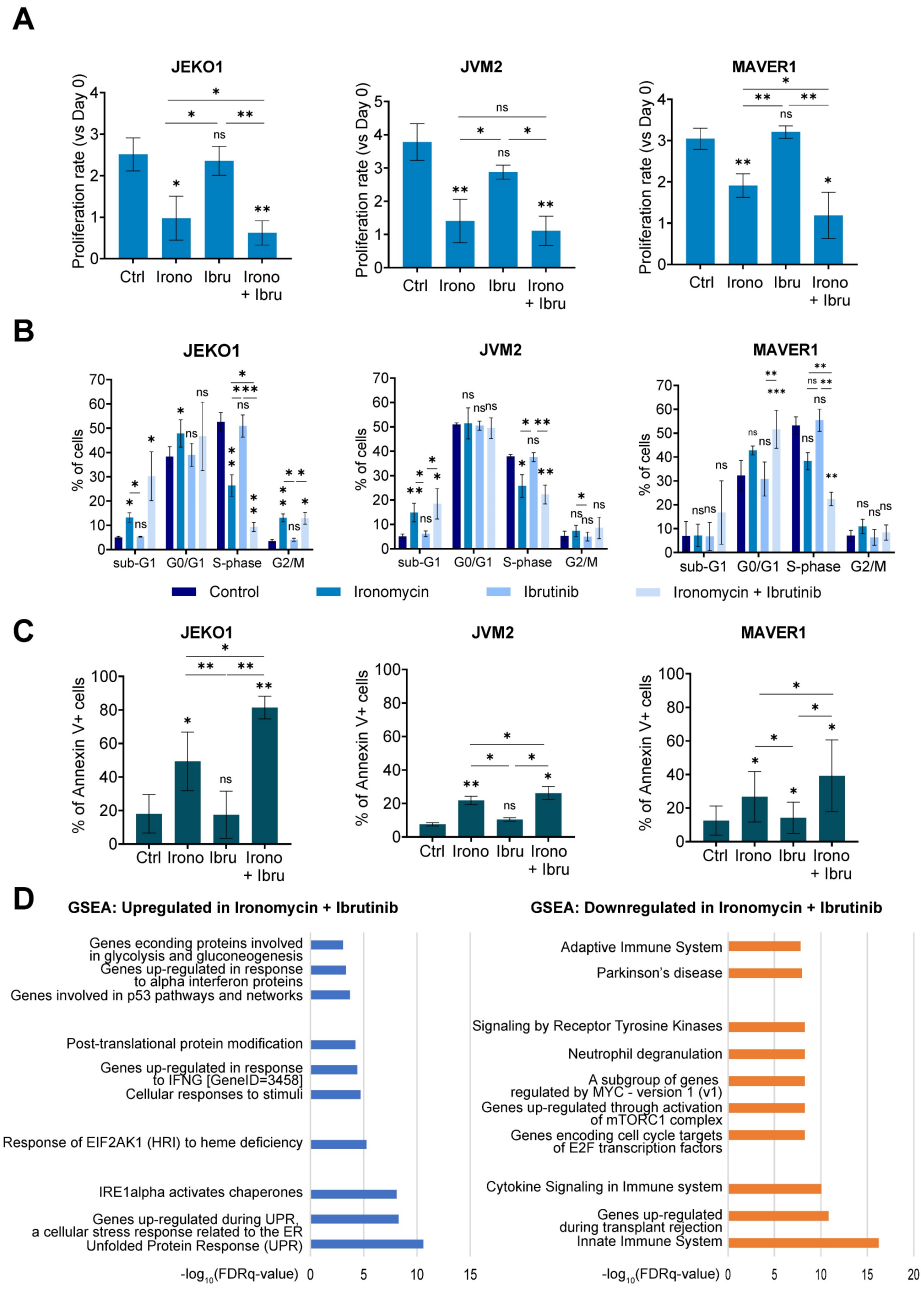
**(A)** JEKO1, JVM2 and MAVER1 cell lines were treated as indicated with ironomycin (JEKO1: 50 nM; JVM2/MAVER1: 250 nM) and ibrutinib (JEKO1: 0.5 μM; JVM2: 1.5 μM; MAVER1: 6.25 μM) for 48 h. Cells were counted at day 0 and at the end of the treatments, and the number of cells was normalized to day 0 to calculate the proliferation rate. Graphs show the mean ± SD of 3 independent experiments. **(B)** Cells were treated as in (A) and BrdU (10 μg/ml) was added during the last 1.5 h of treatment. Cells were fixed and processed to detect BrdU incorporation and total DNA. BrdU+ cells were assigned to S-phase. BrdU- cells were assigned to G0/G1 or G2/M phases based on their DNA content. Results are the mean ± SD of 3-4 independent experiments. **(C)** Cells were treated as in (A) and Annexin V was detected by flow cytometry. Graphs show the mean ± SD of 3-4 independent experiments. (A-C) Asterisks indicate a significant difference compared to control conditions after applying a Student's t-test for pairs. *: p-value < 0.05; **: p-value < 0.01; ***: p- value < 0.001; ****: p-value < 0.0001; ns: not significant. **(D)** Cells were treated as in (A). Total RNA was extracted, RNA-sequencing was performed and GSEA was applied to find upregulated and downregulated pathways in cells treated with ironomycin plus ibrutinib. FDR: false discovery rate.

Furthermore, we observed that the combination of both drugs significantly reduced cell proliferation and induced cell cycle arrest to a greater extent than either drug alone (Figure 5A,B). It also induced strong Cyclin D1 degradation in JEKO1 and moderate in JVM2, together with DNA damage induction (Figure S9A,B). The decrease in proliferation caused by the drug combination correlated with an increase in Annexin V+ cells (Figure 5C). Caspases and PARP cleavage were observed in JEKO1 and MAVER1 (Figure S9C). Moreover, Mcl1 was specifically degraded upon drug combination in JEKO1 cell line, whereas Bcl2 seemed to slightly accumulate in JVM2 (Figure S9D). In order to better understand the molecular mechanism of the synergy, we compared RNA-seq data of cells treated with ironomycin, ibrutinib and the combination of both drugs. At the studied doses, ibrutinib impacted the expression of a low number of genes (22 downregulated and 4 upregulated), but no particular pathway was identified (Table S4). Ironomycin-induced up-regulation of UPR and mTORC signatures was stronger in combination with ibrutinib, especially in JEKO1 and MAVER1 (Figure 5D). Taken together, these results indicate that combination of ironomycin with ibrutinib induces a sustained activation of UPR and a strong inhibition of BCR signaling that trigger toxicity in MCL cells.

## Discussion

Here, we show that targeting iron homeostasis could be of therapeutic interest to target MCL cells, in particular in combination with BTK inhibition. First, using MCL patient data, we identified that deregulation of the expression of iron homeostasis genes can delineate MCL patients with poor outcome (Figure 1A). High expression of genes coding for transferrin receptor (*TFRC*), transcription factor *HIF1-A* (hypoxia induced factor 1A), *APEX1* (APEX endonuclease) and *SLC39A14* was associated to a poor outcome, whereas upregulation of *IREB2* (iron-responsive element binding protein 2), *SCARA3*, *SFXN4* (sideroflexin-4) and *ABCG2* correlated with a good prognosis. These genes were previously reported to be involved in other malignancies, but this is the first study that links six of them to MCL. *TFRC* and *HIF*s are upregulated in many types of cancer, which correlates with poor prognosis and response to therapy 57,58. In particular, elevated *HIF1A* was related to poor prognosis in MCL 59. APEX is activated in response to DNA damage and its dysregulation is associated to several types of cancer 60. *SLC39A14* codes for a metal transporter and was reported downregulated in prostate cancer 61 and upregulated in glioma 62. IREB2 stabilizes the mRNA of *TFRC* and *DMT1* that code for iron transporters, leading to increased intracellular iron concentration 63 and is dysregulated in lung 64 and renal cancers 65. Downregulation of ROS scavenger SCARA3 was reported in prostate cancer 66, hepatocellular carcinoma 67, lung cancer 68 and myeloma 69. Sideroflexin-4 has been suggested as a therapeutic target in ovarian cancer 70. Thus, iron dysregulation is an important feature in cancer biology with various effects depending on the cancer cell type.

The iron chelators deferasirox and deferoxamine are approved by the FDA for treatment of chronic iron overload in patients who are receiving long-term blood transfusions and for conditions such as beta-thalassemia and other chronic anemias 33,71. Regarding their use in cancer treatment, previous pre-clinical studies reported that iron chelation may be of therapeutic interest to treat AML in combination with vitamin D3 72 and triggers the DNA damage response in T-cell acute lymphoblastic leukemia 73. It was reported that deferasirox and vitamin D synergize to promote monocyte differentiation in primary AML cells and prolonged the survival of AML patients 74. Moreover, deferasirox is cytotoxic to lymphoma cells 75, lung cancer cells 76, and multiple myeloma cells 77 among others, and synergizes with gemcitabine to inhibit pancreatic cancer cell growth 78. In addition, other pre-clinical studies using cell lines suggested that deferoxamine or deferasirox may be interesting for MCL treatment 19,34, but none of these agents has been approved for cancer treatment. From a safety point of view, it was reported that treatment with deferasirox presents a risk of kidney failure 79, liver failure 80,81 and gastrointestinal bleeding 82 in some patients. Ironomycin, a synthetic derivate of salinomycin that sequesters iron in the lysosomes and triggers ferroptosis 22, has demonstrated greater efficacy in killing various types of cancer cells compared to iron chelators 20,29, owing to its iron-sequestration specific mechanism of action. In fact, it was described that ironomycin can alter the redox state within lysosomes, increasing ROS production, and induces lysosomal membrane permeabilization, leading to the release of potentially toxic lysosomal enzymes and ROS into the cytosol that can further damage lysosomes and other cellular structures 22.

Our results show that ironomycin is toxic to MCL cells at ~100-fold lower concentrations than deferasirox, suggesting that its side effects if used in cancer therapy would be less than those of deferasirox. In this regard, our previous study using mouse models showed that mice weight was not affected by ironomycin treatment at doses that presented toxicity against DLBCL xenografts 20. Moreover, we found that ironomycin and deferasirox affect primary MCL cells from patients and normal monocytes without inducing toxicity in normal B- and T-lymphocytes (Figure 1). Intriguingly, we observed a small but significant increase in T-lymphocyte percentage upon ironomycin treatment. Given that these cells do not proliferate in our *in vitro* conditions and that iron homeostasis is important for T-lymphocytes 83 , we surmise that dead monocytes may release iron to the medium that may be up taken by the T-lymphocytes in the culture, improving their survival compared to control conditions. Since we only evaluated the global CD3+ T-cell population, further analyses are required to determine which T-lymphocyte sub-population is more abundant and its intracellular iron level upon ironomycin treatment and its impact in *in vivo* models. Using MCL cell lines, we studied the molecular mechanisms of ironomycin cytotoxicity. Chromosome translocation t(11;14) is a genetic hallmark of MCL patients that results in overexpression of Cyclin D1, which is essential to the pathogenesis of this disease by conferring a proliferative advantage to tumor cells 1. In fact, high-risk MCL is associated to the proliferation marker Ki-67 ≥ 30% 84,85. Importantly, we found that ironomycin induces degradation of Cyclin D1 protein, which correlates with a strong decrease in cell proliferation and cell cycle arrest (Figure 2). Our data indicate that Cyclin D1 and D2 down-regulation is due to changes in transcription and increased protein degradation. On the one hand, epigenetic enzymes such as the Jumonji family of histone demethylases or the DNA Ten-Eleven Translocation (TET) methylcytosine dioxygenases have been reported to depend on iron as a co-factor 12. Thus, iron depletion caused by ironomycin would have an impact on epigenetic and transcriptional regulation through these enzymes. Moreover, our results show that ironomycin activates an UPR characterized by the accumulation of IRE1α (Figure 4B). IRE1α is responsible for the regulated IRE1α-dependent decay (RIDD) that cleaves selected mRNAs, decreasing the proteins that they code for 86. Thus, it is possible that constitutive UPR activation and IRE1α accumulation lead to degradation of mRNA coding for Cyclin D1. On the other hand, UPR activation characterized by p-eIF2α like in JVM2 and MAVER1 (Figure 4B) induced by ironomycin can also lead to translation attenuation, which will eventually reduce Cyclin D1 and Cyclin D2 levels due to protein turnover coupled to lack of new protein synthesis. Mutation or deletion of *TP53*, which is a major cell cycle regulator, is related to high-risk disease 87,88. Interestingly, our data show that ironomycin triggers apoptosis in the three MCL cell lines independently of their *TP53* status (Table S2). These findings strengthen the potential of targeting iron homeostasis as a way to impair MCL cells growth and slow down tumor progression, even in *TP53* dysregulated patients.

We observed that ironomycin induced changes in the abundance of pro-apoptotic and anti-apoptotic proteins of the Bcl-family. In JEKO1, the anti-apoptotic protein Mcl1 was the main factor degraded, which explains the triggering of apoptosis. In JVM2/MAVER1, upon ironomycin treatment, all studied factors accumulated regardless of whether they were pro- or anti-apoptotic. It was published that Bak interacts with Mcl1 and that disrupting this interaction induces Mcl1 degradation 89. Bax expression is regulated by the tumor suppressor p53 and has been shown to be involved in p53-mediated apoptosis. The association and the ratio of Bax to Bcl2 also determines survival or death of a cell following an apoptotic stimulus. In JVM2, which expresses wild type p53, we observed a significant increase in Bax, Bcl-xL, Bcl2 and Mcl1 levels. Our BH3-profiling data show that JVM2 is mostly dependent on Bcl2, with lower dependence on Mcl1 or Bcl-xL. In MAVER1, Bax and Bcl2 expression inversely correlated, maybe due to a lack of functional p53 in this cell line. Our BH3-profiling assay confirmed a greater dependence of MAVER1 on Bcl2, suggesting that the slight increase in Bax observed by western blot is not sufficient to efficiently trigger caspase-dependent apoptosis as observed in our other data (Figure S2). In addition, upon DNA damage caused by ROS, anti-apoptotic proteins like Bcl2 and Bcl-xL can be upregulated or activated in an attempt to delay or prevent apoptosis, allowing the cell to repair the damage. If the damage is irreparable, the pro-apoptotic signals may override the anti-apoptotic mechanisms, leading to cell death.

In addition, ironomycin induced ROS production, lipid peroxidation, DNA damage and sustained UPR activation, leading to apoptosis and ferroptosis. Of note, these effects were achieved using nanomolar concentrations of ironomycin, in contrast to deferasirox which exhibited cytotoxicity at concentrations 10-100 times higher, suggesting that ironomycin could be used at low dose to minimize toxicity and side effects. The toxicity of ironomycin was already investigated in mice and did not underline significant toxicities in the range of doses deleterious for cancer cells 20,22,29. Moreover, iron supplementation was able to rescue cell death caused by deferasirox, but not by ironomycin, indicating that the cytotoxicity of ironomycin is not due to limited iron availability for metabolic and enzymatic reactions and therefore its therapeutic potential diverges from that of iron chelators. We previously reported the efficacy of ironomycin in targeting B-lymphoma cells using a syngeneic A20 murine model 20. Further investigation using specific PDX models is needed to determine optimal ironomycin doses to kill MCL cells and to assess toxicity *in vivo*.

Ironomycin was described to induce ferroptosis by causing lipid peroxidation. We analyzed the contribution of lipids to ironomycin cytotoxicity and found that lipid deprivation in culture medium had different effects depending on the cell line (Figure S6). Lack of exogenous lipids only increased the toxicity of ironomycin in JVM2 cells (Figure S6A) which does not present TAG and sterol esters accumulated in lipid droplets. This result suggests that the capacity of JVM2 to cope with lipid peroxidation is mostly dependent on its ability to uptake lipids from the medium to substitute the oxidized ones, since its intracellular lipid stock is low. The cytotoxicity of ironomycin on JEKO1 and MAVER1 was not affected by the lack of exogenous lipid source. Both cell lines present lipids stored in lipid droplets, which number and size was decreased upon ironomycin treatment (Figure S6E), probably due to the use of those stored lipids to try and repair the ROS-damaged membranes. These results point at the importance of lipids as targets of ironomycin toxicity and raise the question of how lipid metabolism could impact the response to drugs targeting iron homeostasis. It has already been reported that lipid metabolism modulates the DNA damage response 90, which can impact cell response to chemotherapy. Since pharmacological and dietary manipulations of lipids are possible, it would be interesting to assess a potential synergy between decreasing the pool of lipids and targeting iron homeostasis as a therapeutic strategy to kill cancer cells.

It has been described that sustained ER-stress causes the UPR to trigger apoptosis 91. Interestingly, we found that ironomycin upregulates the UPR, notably related to IRE1α accumulation and activation. The proteasome inhibitor bortezomib, currently under clinical investigation in MCL, similarly activates an apoptotic UPR in multiple myeloma 92, suggesting that targeting iron in combination with proteasome inhibitors may hold therapeutic promise. IRE1α has kinase and RNAse activities 93 and produces the spliced form XBP1s that targets genes coding for proteins that enhance protein folding capacity and quality control 94. High activation of IRE1α can also cleave other mRNAs with similar structure to that of XBP1, causing apoptosis 86. IRE1α activates the apoptotic signaling kinase 1 (ASK1), which in turn triggers downstream factors such as JNK and p38 MAPK, enhancing apoptosis. In addition, it has been shown that persistent ER stress produces ROS 95. These notions raise the idea that ironomycin induced-ROS production leads to ER stress and UPR activation that will in turn produce more ROS, creating an amplification loop culminating in apoptosis. Moreover, there is increasing evidence of a link between UPR, in particular IRE1α, and lipid metabolism regulation 96. Ironomycin caused lipid peroxidation, which must be replaced by new lipids to maintain membrane integrity. ER regulates lipid synthesis and is itself tightly regulated by UPR 97, which may explain UPR activation and IRE1α accumulation upon ironomycin-dependent lipid peroxidation. Interestingly, it has also been reported that IRE1α can trigger mitochondrial (intrinsic) apoptosis in a Bax/Bak-dependent manner 91 and ironomycin triggers a non-canonical Bax/Bak-dependent apoptosis in AML 29. Our BH3 profiling experiments show that ironomycin changes the dependencies of MCL cells to Blc2-family anti-apoptotic factors and induces changes in BAX expression (Figure 3C,D and Figure S3). We found a synergy between iron dysregulation and inhibitors of Bcl2-family anti-apoptotic factors which could be of therapeutic interest. Moreover, we proved that ironomycin caused significant changes in basal and maximal mitochondrial respiratory capacities and reduced ATP production (Figure S4C,D). Altogether, these results indicate that ironomycin exerts profound toxicity on mitochondria, triggering apoptosis in MCL cells, as well as ferroptosis linked to ROS production and lipid peroxidation. Unlike in DLBCL cells 20, ironomycin seemed to not cause ferritinophagy in all MCL cell lines, suggesting that malignant B-cells from diverse origins exhibit distinct vulnerabilities related to iron metabolism.

**Figure 6:**
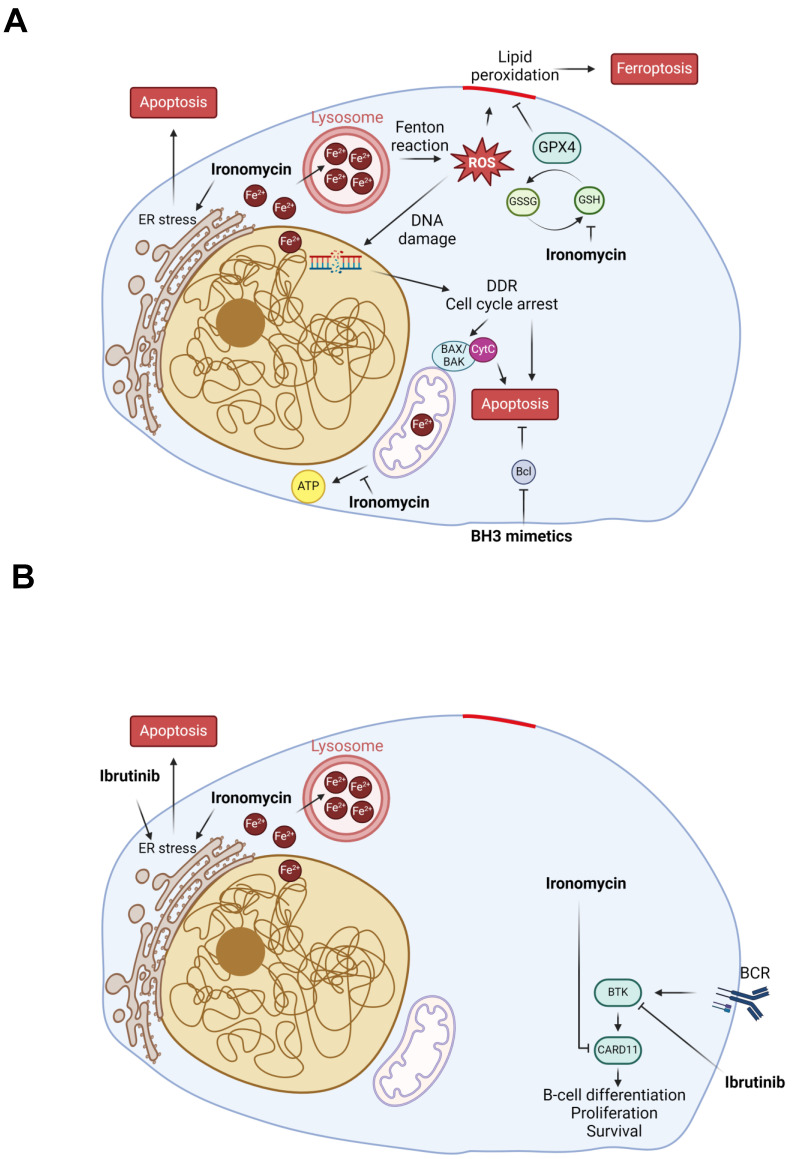
**Model of ironomycin cytotoxic effects alone and in combination with other drugs**. **(A)** Ironomycin sequesters iron in lysosomes triggering different cellular responses: (1) the production of ROS through the Fenton reaction that cause peroxidation of lipids, which require GPX4 activity to be detoxified, and DNA damage that will cause cell cycle arrest; (2) impairment of mitochondrial metabolism and ATP production; (3) ER stress characterized by the activation of UPR, notably the IRE1α signaling pathway. High levels of lipid peroxidation, DNA damage, mitochondrial activity impairment and sustained ER stress lead to ferroptosis and apoptosis. Combination of ironomycin with BH3 mimetics have a synergistic toxic effect in MCL cells. **(B)** Ironomycin downregulates BCR-signaling and synergizes with ibrutinib. Combination of both drugs further increases a sustained UPR that leads to apoptosis. Figures were created with Biorender.com.

Finally, MCL is characterized by aberrant activation of the BCR pathway, which is initiated by BCR stimulation and BTK activation to regulate the downstream NF-κB and PI3K/AKT/mTOR pathways. Thus, BTK inhibitors such as ibrutinib are used in relapse/refractory MCL patients with good initial response 98 and the benefit of BTK inhibitors use earlier in the treatment course is under investigation with encouraging results 8,99-102. However, resistance to ibrutinib is very frequent and new strategies to overcome it using drug combination are being explored 8,99-102 (ENRICH clinical trial: ISRCTN11038174). It has been suggested that B-cells resistance to ibrutinib can have different origins including gene mutation, transcriptional dysregulation or tumor microenvironment mediation 103. Through RNA-seq, we found that ironomycin downregulates a BCR signature and confirmed the reduction of CARD11 protein, a BCR pathway downstream factor. CARD11 gain-of-function was also shown to induce BCL2A1 expression and promote drug resistance in MCL 104. This prompted us to investigate the combination of ironomycin with ibrutinib, which synergized to kill MCL cells even in ibrutinib-resistant MAVER1 cell line. Our data indicate that ironomycin and ibrutinib synergize to impair MCL cells proliferation and cause sustained elevated UPR activation incompatible with cell survival. Moreover, combination of venetoclax and ibrutinib to treat relapse/refractory MCL patients showed a remission rate of 71% 6; however, resistance to this drug combination has been reported 105. Currently, an ongoing phase 3 clinical trial (SYMPATICO: #NCT03112174) is evaluating the combination ibrutinib plus venetoclax vs ibrutinib alone in relapsed MCL patients. We observed a synergy of ironomycin with both venetoclax and ibrutinib (Figure S3 and Figure 4), suggesting that targeting iron homeostasis could be a promising strategy for patients who develop drug resistance. The mechanisms of ironomycin effect alone and in combination with other drugs analyzed in this study, namely ibrutinib and Bcl2-family inhibitors, are summarized in the model in Figure 6. Altogether, our findings underscore the therapeutic potential of targeting iron homeostasis to overcome drug resistance in MCL.

## Supplementary Material

Supplementary figures and methods.

Supplementary table 1.

Supplementary tables 2-3.

Supplementary table 4.
