# Supplementary material for: Ironomycin induces mantle cell lymphoma cell death by targeting iron metabolism addiction
Source: Theranostics. 2025 Feb 3;15(7):2834–51. doi: 10.7150/thno.101821 (PMC11898298; doi:10.7150/thno.101821)
Supplement: Supplementary file 1 — Supplementary figures and methods. [file thnov15p2834s1.pdf]

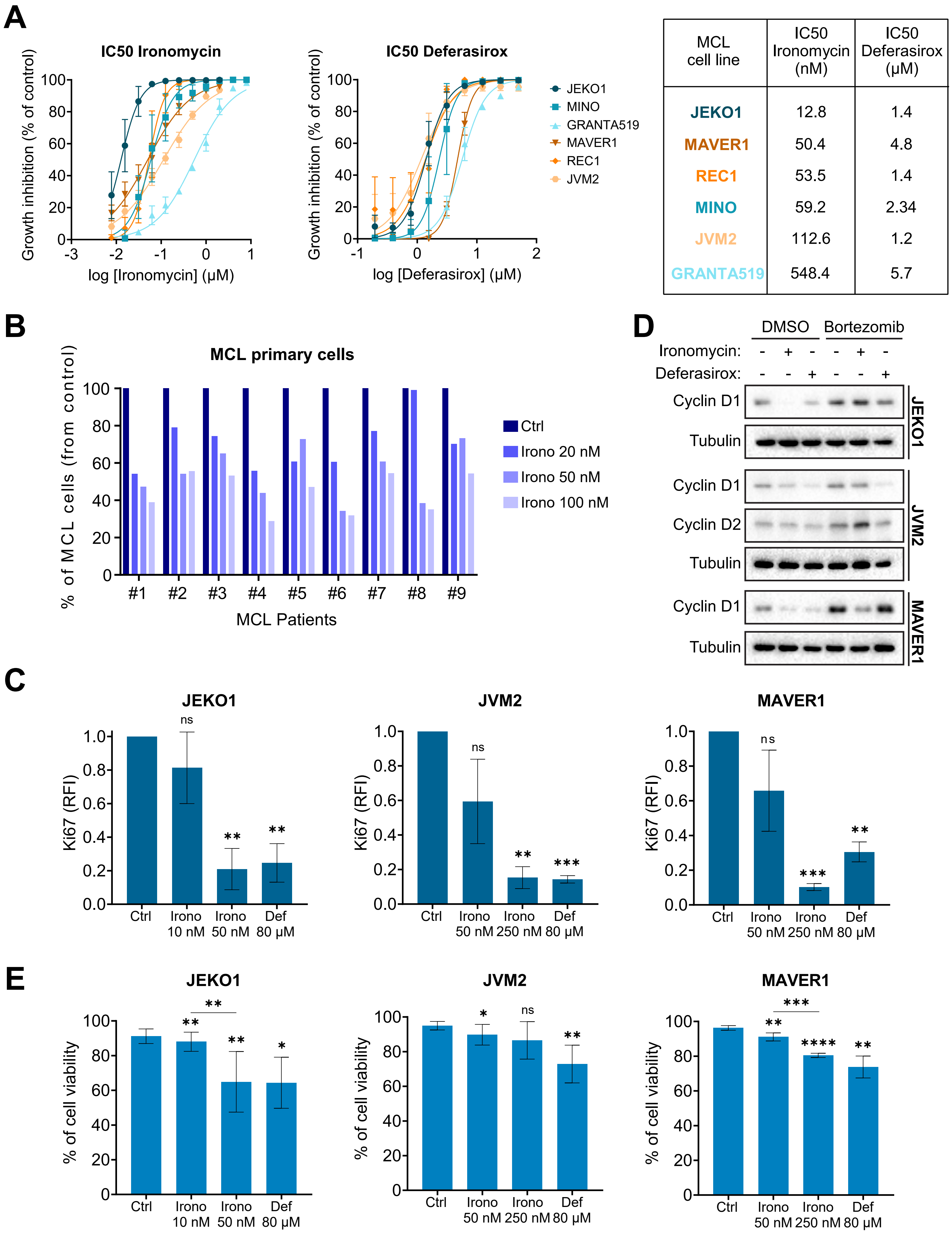
A
IC50 Ironomycin
Growth inhibition (% of control)
log [Ironomycin] (µM)
IC50 Deferasirox
log [Deferasirox] (µM)
JEKO1
MINO
GRANTA519
MAVER1
REC1
JVM2
MCL cell line | IC50 Ironomycin (nM) | IC50 Deferasirox (µM)
JEKO1 | 12.8 | 1.4
MAVER1 | 50.4 | 4.8
REC1 | 53.5 | 1.4
MINO | 59.2 | 2.34
JVM2 | 112.6 | 1.2
GRANTA519 | 548.4 | 5.7
B
MCL primary cells
% of MCL cells (from control)
Ctrl
Irono 20 nM
Irono 50 nM
Irono 100 nM
#1 #2 #3 #4 #5 #6 #7 #8 #9
MCL Patients
C
JEKO1
JVM2
MAVER1
Ki67 (RFI)
Ctrl
Irono 10 nM
Irono 50 nM
Irono 250 nM
Def 80 µM
ns
D
DMSO
Bortezomib
Ironomycin:
Deferasirox:
Cyclin D1
Cyclin D2
Tubulin
JEKO1
JVM2
MAVER1
E
JEKO1
JVM2
MAVER1
% of cell viability

Supplementary Figure S2

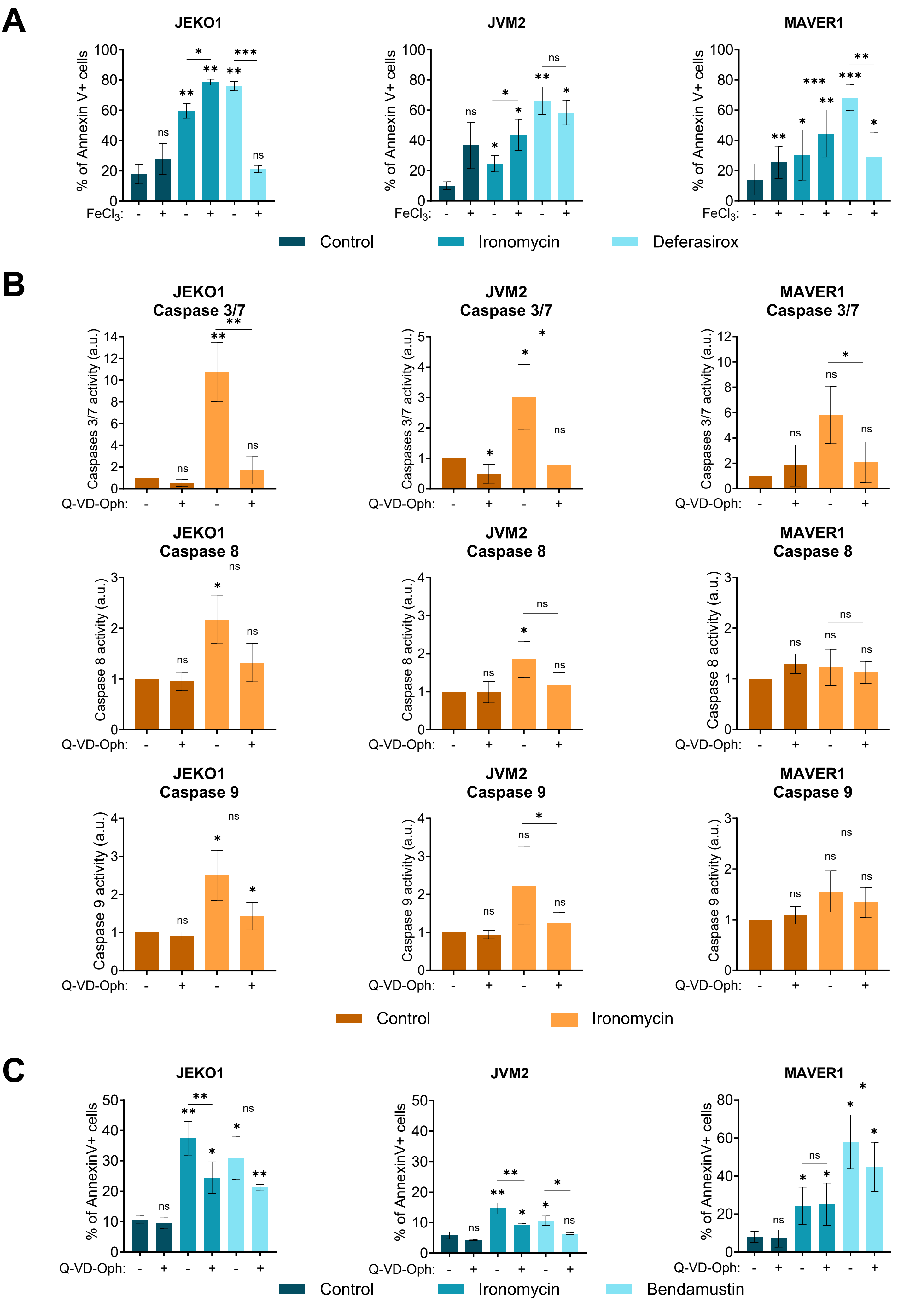

Supplementary Figure S3

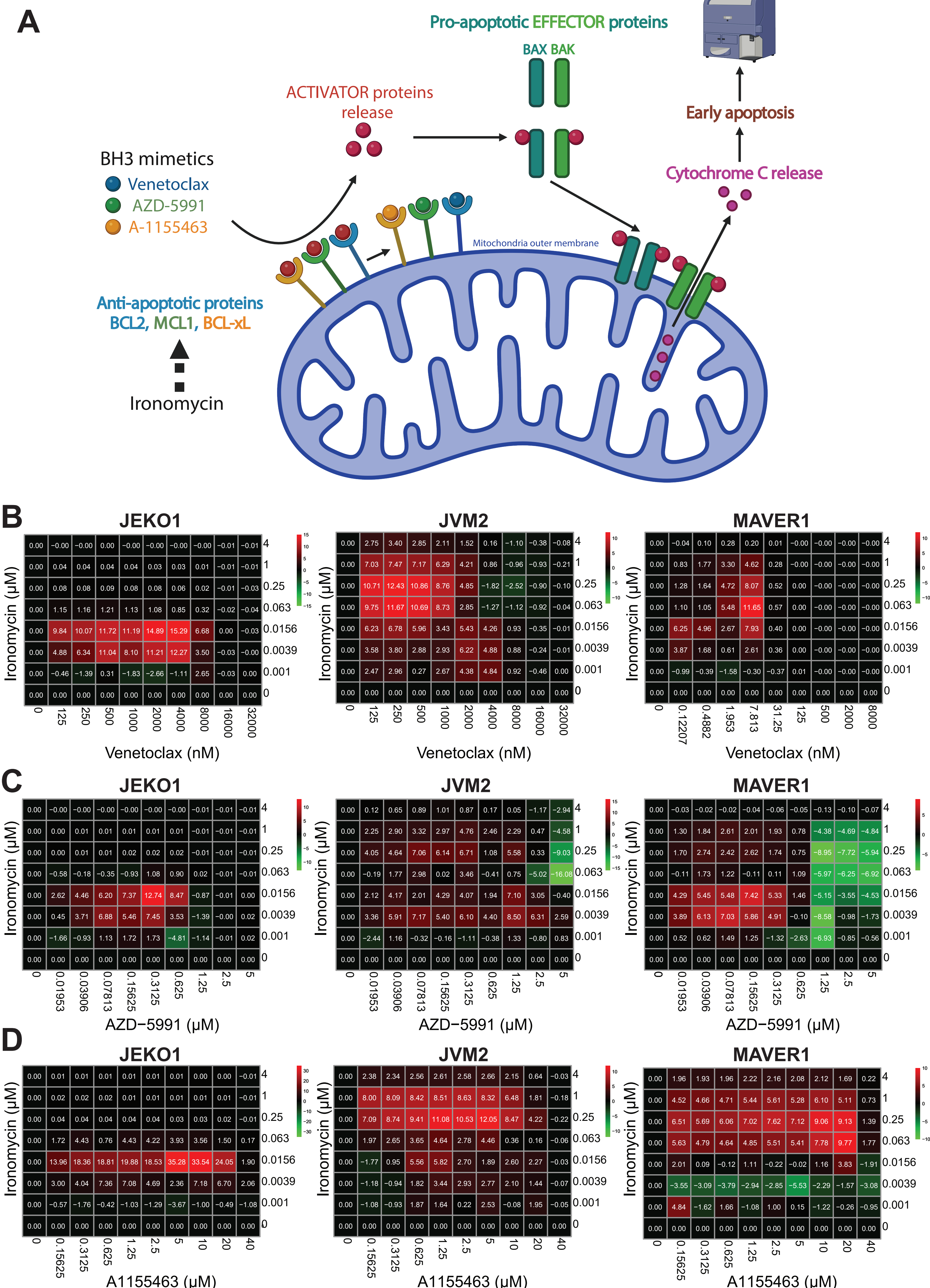

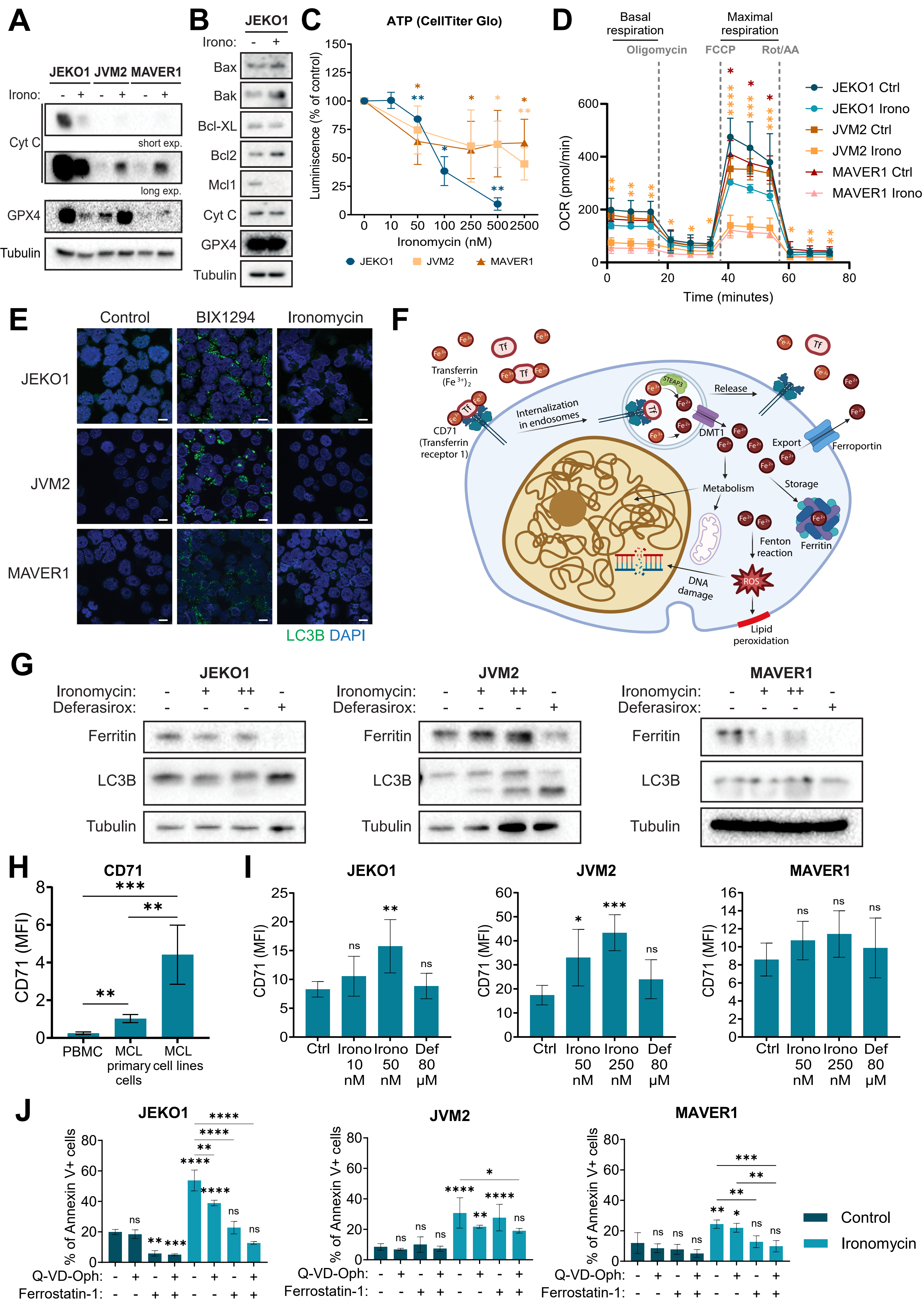

A
JEKO1 JVM2 MAVER1
Irono:
Cyt C
short exp.
long exp.
GPX4
Tubulin
B
JEKO1
Irono:
Bax
Bak
Bcl-XL
Bcl2
Mcl1
Cyt C
GPX4
Tubulin
C
ATP (CellTiter Glo)
Luminiscence (% of control)
Ironomycin (nM)
JEKO1
JVM2
MAVER1
D
Basal respiration
Maximal respiration
Oligomycin
FCCP
Rot/AA
OCR (pmol/min)
Time (minutes)
JEKO1 Ctrl
JEKO1 Irono
JVM2 Ctrl
JVM2 Irono
MAVER1 Ctrl
MAVER1 Irono
E
Control
BIX1294
Ironomycin
JEKO1
JVM2
MAVER1
LC3B DAPI
F
Transferrin (Fe3+)2
CD71 (Transferrin receptor 1)
Internalization in endosomes
STEAP3
Release
DMT1
Export
Ferroportin
Metabolism
Storage
Ferritin
Fenton reaction
ROS
DNA damage
Lipid peroxidation
G
JEKO1
JVM2
MAVER1
Ironomycin:
Deferasirox:
Ferritin
LC3B
Tubulin
H
CD71
CD71 (MFI)
PBMC
MCL primary cells
MCL cell lines
I
JEKO1
JVM2
MAVER1
CD71 (MFI)
Ctrl
Irono 10 nM
Irono 50 nM
Irono 250 nM
Def 80 µM
J
JEKO1
JVM2
MAVER1
% of Annexin V+ cells
Q-VD-Oph:
Ferrostatin-1:
Control
Ironomycin

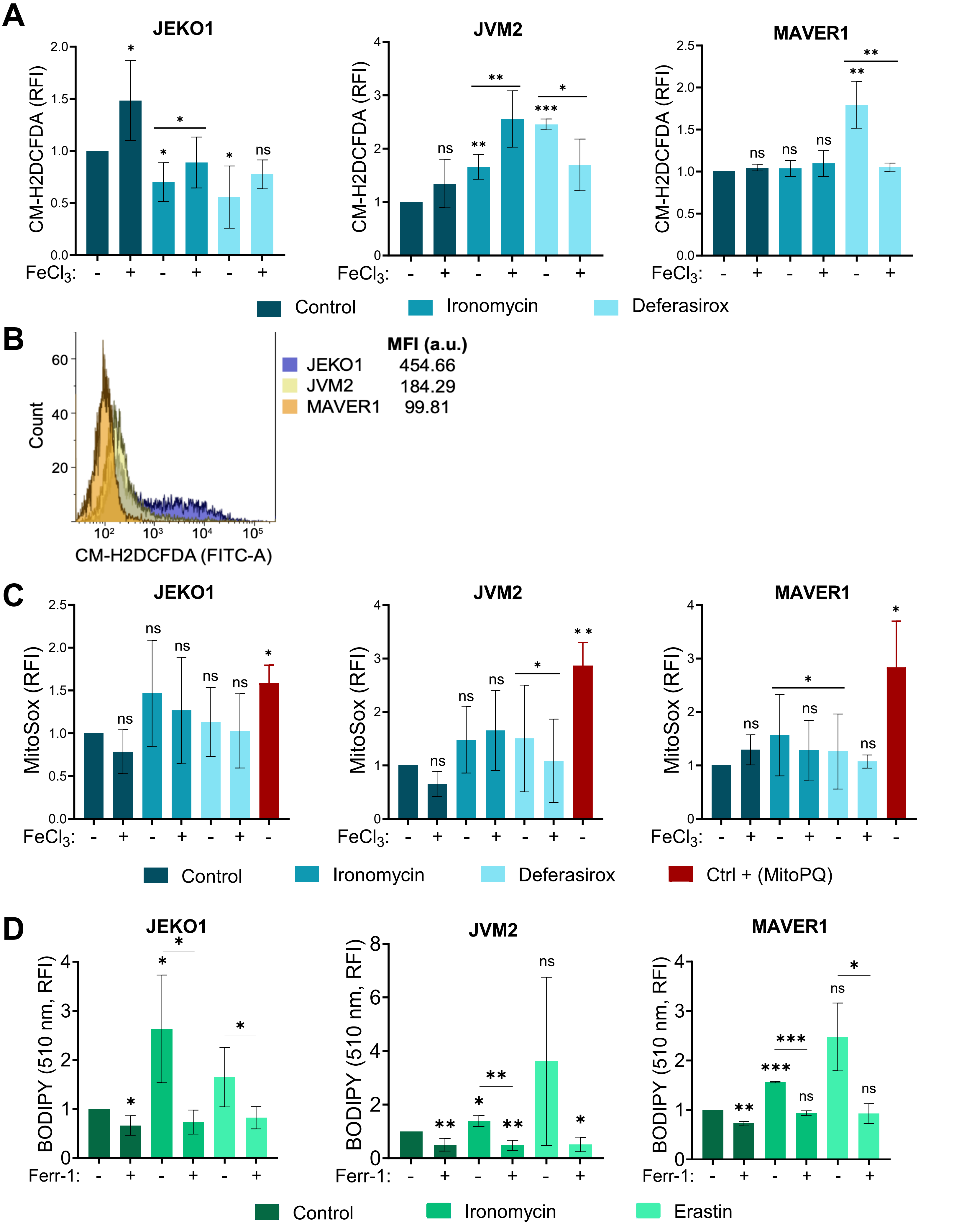

A
JEKO1
JVM2
MAVER1
CM-H2DCFDA (RFI)
FeCl3:
Control
Ironomycin
Deferasirox
B
Count
CM-H2DCFDA (FITC-A)
MFI (a.u.)
JEKO1 454.66
JVM2 184.29
MAVER1 99.81
C
MitoSox (RFI)
Ctrl + (MitoPQ)
D
BODIPY (510 nm, RFI)
Ferr-1:
Erastin

Supp Figure S6

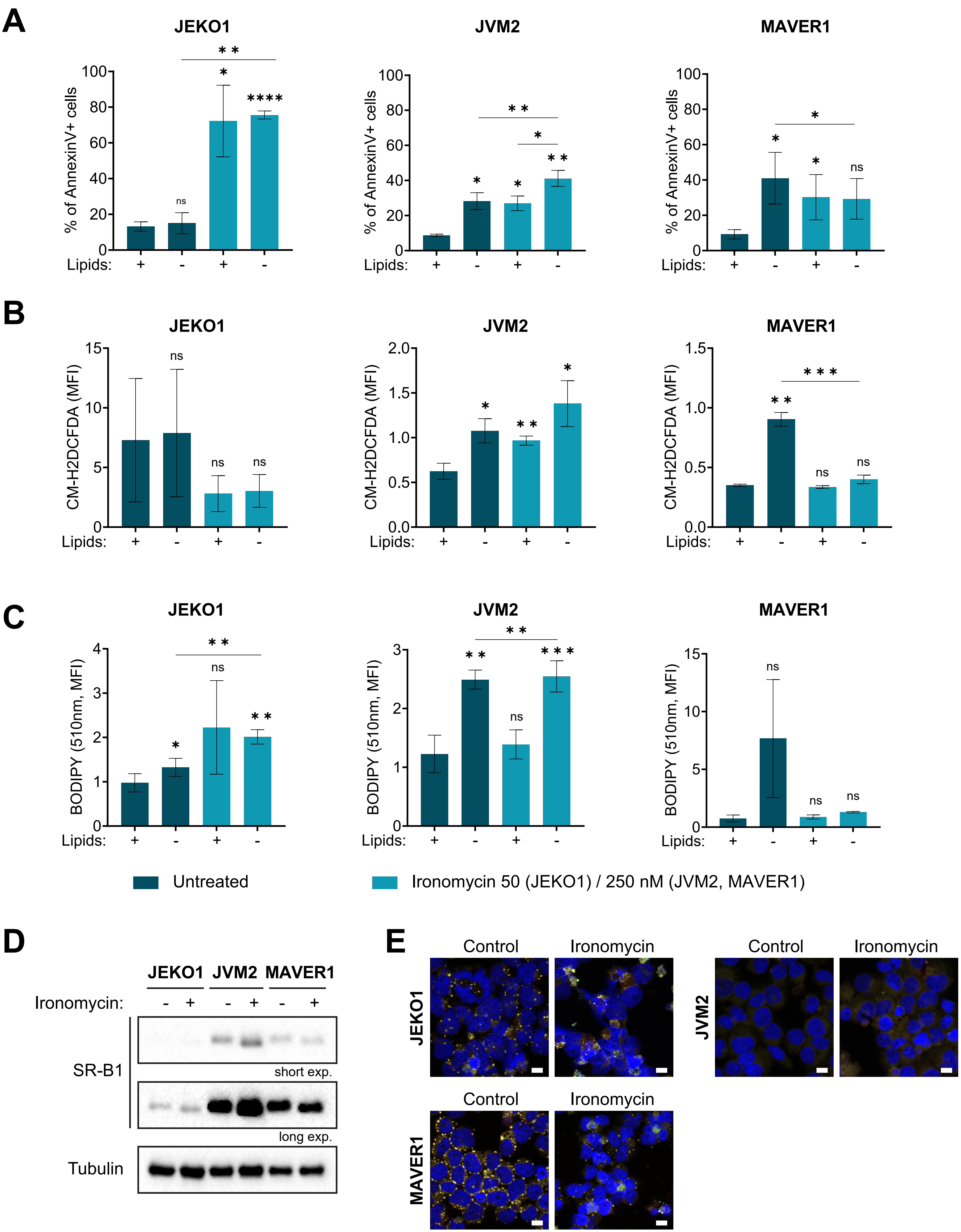

Supplementary Figure S7

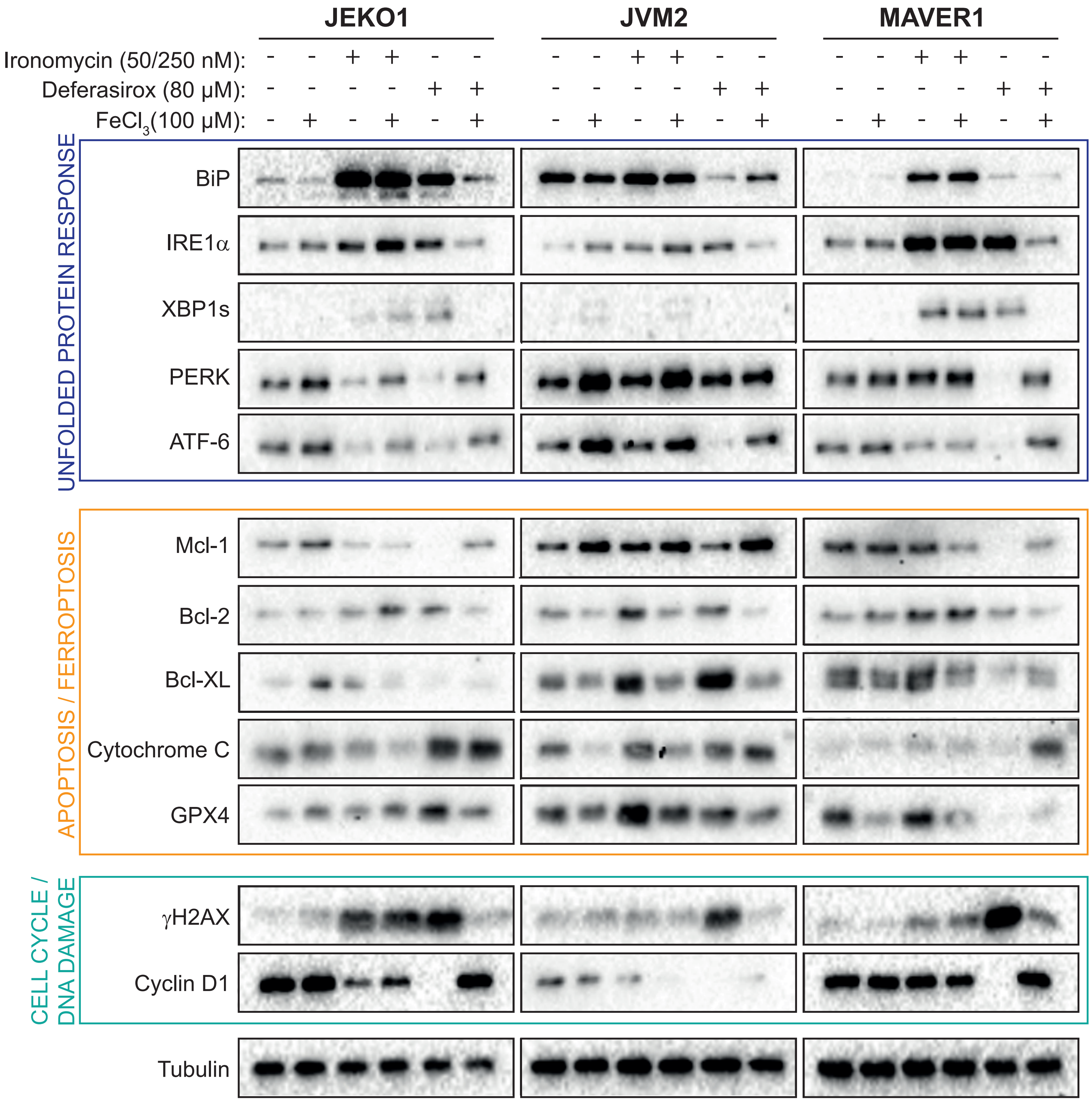

Supplementary Figure S8

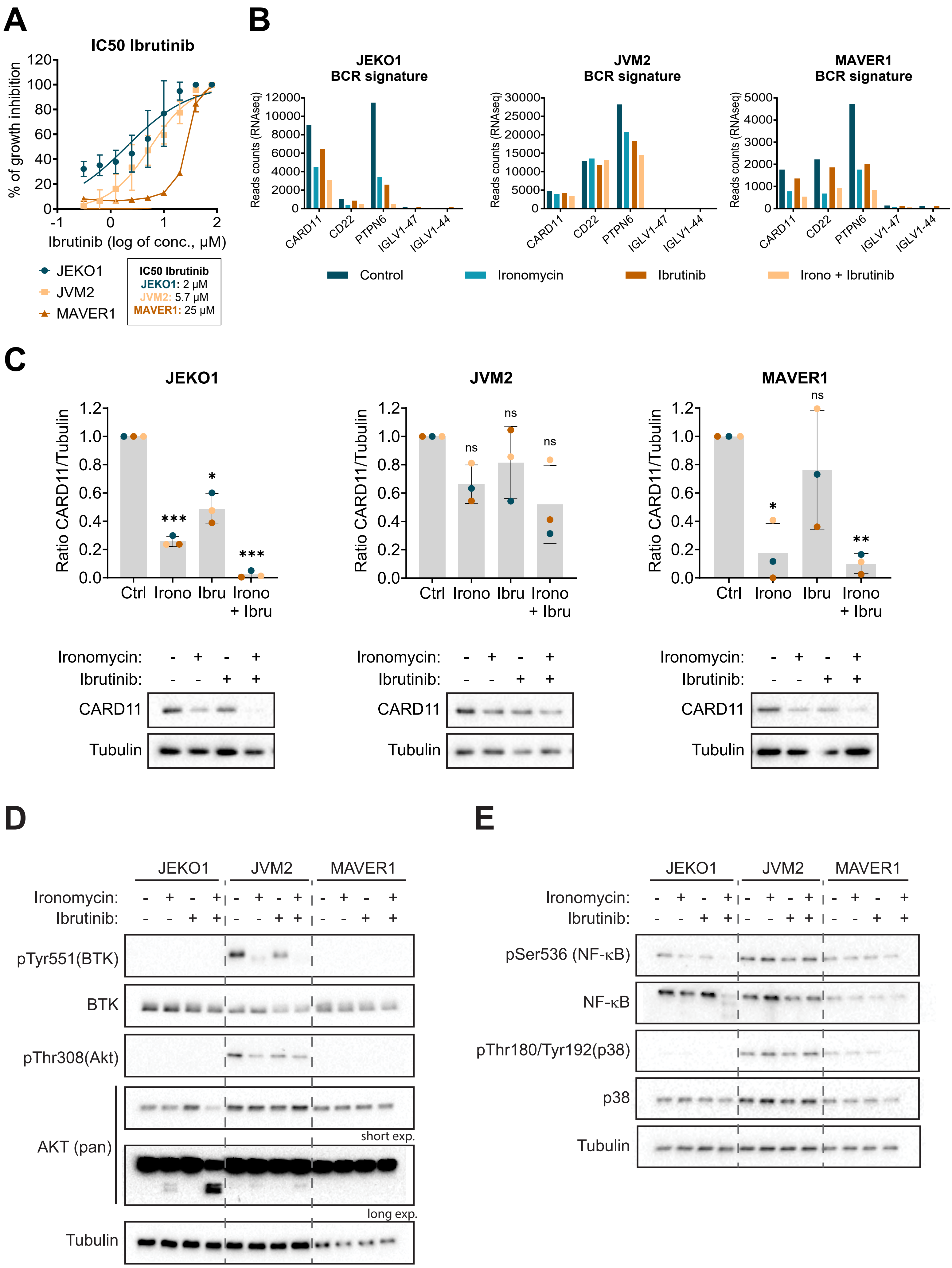

Supplementary Figure S9

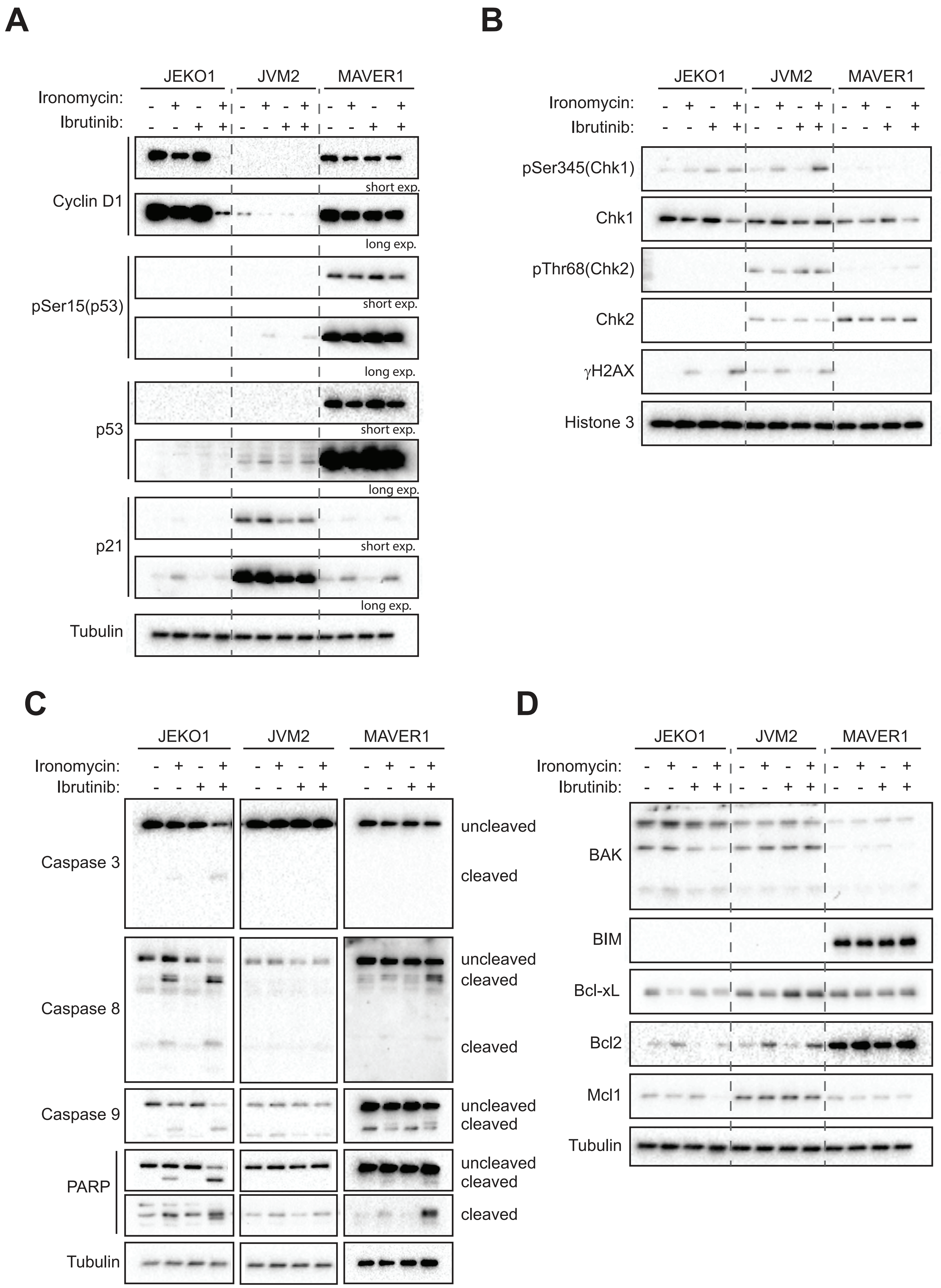

Supplementary Figure S1. (A,B) A panel of 6 MCL cell lines was treated with increasing concentrations of (A) ironomycin (range: 0.0078 – 8 µM) or (B) deferasirox (range: 0.195 – 50 µM) for 4 days. At day 4, cell viability was assessed using CellTiter-Glo Luminescent Cell Viability Assay. IC50 for each cell line was calculated using GraphPrism software. Left and middle panels show the nonlinear regression curve of growth inhibition for all MCLs. Right panel shows the IC50 value for each drug and each MCL cell line. Data are based on at least 3 independent experiments. **(B)** Primary MCL cells from 9 patients were treated with ironomycin at the indicated concentrations for 4 days. Tumor cells were analyzed by flow cytometry and expressed in % of control. Graphs shows the individual response of each patient used to build the graph in Figure 1C. **(C)** Cells were treated as indicated for 2 days and processed to detect the proliferation marker Ki67 with a specific antibody by flow cytometry. RFI: relative fluorescence intensity (normalized to control conditions). Results are the mean ± SD of 3 independent experiments. Asterisks indicate a significant difference compared to control conditions after applying a Student's t-test for pairs. **: p-value < 0.01; ***: p: value < 0.001; ns: not significant. **(D)** Cells were pre-treated with bortezomib (BTZ, 5 µM) for 1 h and ironomycin (JEKO1: 1 µM; JVM2/MAVER1: 5 µM) and deferasirox (80 µM) were added for 6 h. Cells were collected and lysed in Laemmli buffer to perform western blotting to analyze the levels of Cyclin D1 and Cyclin D2. Tubulin was used as a loading control. Figure shows 1 representative out of 3 independent experiments. **(E)** Cells were treated as indicated for 2 days, counted in presence of trypan blue to visually distinguish dead cells (trypan blue positive) from living cells (trypan blue negative). Viability was calculated as the percentage of living cells to total cells (living + dead). Results are the mean ± SD of at least 4 independent experiments. Asterisks indicate a significant difference compared to control conditions after applying a Student's t-test for pairs. *: p-value < 0.05; **: p-value < 0.01; ***: p-value < 0.001; ****: p-value < 0.0001; ns: not significant.

Supplementary Figure S2. (A) Cells were treated with ironomycin (JEKO1: 50 nM; JVM2/MAVER1: 250 nM) or the iron chelator deferasirox (80 µM). Four hours later, $FeCl_3$ (100 µM) was added to the indicated samples as an exogenous source of iron, and cells were incubated for 48 h. Annexin V was detected by flow cytometry. Results are the mean ± SD of at least 3 independent experiments. Asterisks indicate a significant difference compared to control conditions after applying a Student's t-test for pairs. *: p-value < 0.05; **: p-value < 0.01; ***: p- value < 0.001; ns: not significant. **(B)** Cells were pre-incubated for 30 min with the pancaspase inhibitor Quinoline-Val-Asp-Difluorophenoxymethylketone (Q-VD-Oph, 20 µM) before treatment with ironomycin (JEKO1: 50 nM; JVM2/MAVER1: 250 nM) or bendamustin (20 µM) for 48 h. At the end of the treatments, the activities of caspases 3/7, caspase 8 and caspase 9 were analyzed using the corresponding CaspaseGlo® Assay. The results were normalized to control conditions in each case. Results show the mean ± SD of 3-4 independent experiments. a.u.: arbitrary units. **(C)** Cells were treated with Q-VD-Oph and Ironomycin as in (A). High dose of the alkylating agent bendamustin (20 µM) was used as a positive control to induce cell death. Annexin V was detected by flow cytometry. Results show the mean ± SD of 3 independent experiments. Asterisks indicate a significant difference compared to control conditions after applying a Student's t-test for pairs. *: p-value < 0.05; **: p-value < 0.01; ns: not significant.

Supplementary Figure S3. (A) Schematic view of BH3 profiling principle. BH3 dynamic profiling technique allows measurement of the variation in the anti-apoptotic dependencies of cells after treatment. Specific BH3 mimetics (Venetoclax for Bcl2, AZD-5991 for Mcl1 and A-1155463 for Bcl-xL) are used to measure MCL cells anti-apoptotic dependencies with ironomycin treatment. Each BH3 mimetic competes for anti-apoptotic proteins catalytic pocket, thus releasing activator proteins which in turn activate pro-apoptotic effector proteins. Those proteins will create pores

in the mitochondrial outer membrane allowing the release of Cytochrome C, which is the first step of apoptosis. This phenomenon is detected by staining the cells with an Annexin V and 7AAD to measure the early apoptosis cells by flow cytometry. **(B-D)** Cells were seeded in 96-flat bottom plates and treated with increasing concentrations of ironomycin (range: 0.001 – 4 µM) combined with (B) venetoclax (range: 0.125 – 32 µM), (C) AZD-5991 (range 0.02 – 5 µM), and (D) A1155463 (range: 0.156 – 40 µM). At day 4, cell viability was assessed using CellTiter-Glo Luminiscent Cell Viability Assay. Drug synergy was calculated using R package "SynergyFinder". Effect of drug combination on cell growth is shown in a pseudo-color scale from red (synergism) to green (antagonism). Matrixes show the mean of 3-4 independent experiments.

Supplementary Figure S4. (A) Cells were treated as in Figure 2F and the levels of Cytochrome C and GPX4 were analyzed by western blot. Tubulin was used as loading control. Figure shows 1 representative out of 3 independent experiments. **(B)** JEKO1 cells were treated as in Fig 2F and dead cells were removed by Ficoll centrifugation prior to western blot analysis. Tubulin was used as loading control. Figure shows 1 representative out of 3 independent experiments. **(C)** Cells were treated with the indicated concentrations of ironomycin for 2 days. At the end of the treatment, cells were counted, resuspended at $5x10^5$ cells/ml and 50 µl were deposited in triplicates in opaque 96-well plates. Immediately, 50 ul of CTG were added to each well to measure cellular ATP showed as luminescence and expressed as percentage of the signal in control conditions for each cell line. Results are the mean ± SD of 3 independent experiments. **(D)** Cells were treated with Ironomycin (JEKO1: 25 nM; JVM2/MAVER1: 250 nM) for 2 days, counted, resuspended in warmed-assay medium at $4x10^6$ cells/ml and 50 µl were deposited in triplicates in cell-tak precoated 96-well plate. The mitochondria respiration activity was then assessed by seahorse mito-stress assay. Results are the mean ± SD of 3 independent experiments. **(E)** Cells were treated with ironomycin (JEKO1: 50 nM; JVM2/MAVER1: 250 nM) and BIX1294 (2 µM) for 2 days, deposited onto slides by cytospin centrifugation, fixed with 4% PFA (10 min, RT) and IF was performed to detect LC3B foci. DNA was stained with DAPI. Scale bar = 10 µm. **(F)** Ferric-iron (Fe^{3+}) binds to transferrin, and this complex will be recognized by the transferrin receptor (Trf1 or CD71) for its internalization into the cell. Ferric-iron will be captured into acidified endosomes, released from transferrin and STEAP3 will transform it into ferrous-iron (Fe^{2+}), which is the biologically active form in the cells, necessary for cell functions (DNA synthesis/repair, mitochondrial metabolism, cofactor of numerous enzymes, among others). Ferrous-iron will be released to the cytosol by DMT1. In normal conditions, Fe^{2+} excess is excreted from the cells by ferroportin. Excessive intracellular Fe^{2+} accumulation can be dangerous as it is the substrate for the Fenton reaction, which induces the production of reactive oxygen species (ROS), which cause DNA damage and lipid peroxidation, that are highly toxic to the cell. Image created with BioRender.com **(G)** Cells were treated with ironomycin (JEKO1: 10/50 nM; JVM2/MAVER1: 50/250 nM) and deferasirox (80 µM) for 2 days, collected and whole cell lysates were analyzed by western blot. Tubulin was used as loading control. Figure shows one representative of 3 independent experiments. **(H)** Membrane-bound CD71 level was analyzed by flow cytometry in PBMC from healthy donors (n = 3), primary MCL cells from patients (n = 4) and MCL cell lines (n =6). Asterisks indicate a significant difference compared to control conditions after applying a Student's t-test for pairs. **: p-value < 0.01; ***: p-value < 0.001. MFI: median fluorescence intensity. **(I)** Cells were treated as indicated for 48 h and membrane-bound CD71 level was analyzed by flow cytometry. Results show the mean ± SD of at least 3 independent experiments. Asterisks indicate a significant difference compared to control conditions after applying a Student's t-test for pairs. *: p-value < 0.05; **: p-value < 0.01; ***: p-value < 0.001; ****: p-value < 0.0001; ns: not significant. **(J)** Cells were pre-treated with the ferroptosis inhibitor

Ferrostatin-1 (10 µM, 30 min) or pancaspase inhibitor Q-VD-Oph (20 µM) before treatment with ironomycin (JEKO1: 50 nM; JVM2/MAVER1: 250 nM) or the ferroptosis inducer erastin (4 µM) for 48 h. Annexin V was detected by flow cytometry. Results are the mean of 3-4 independent experiments. Asterisks indicate a significant difference compared to control conditions after applying a two-way ANOVA test. *: p-value < 0.05; **: p-value < 0.01; ***: p-value < 0.001; ****: p-value < 0.0001; ns: not significant.

Supplementary Figure S5. (A,B) Cells were with ironomycin (JEKO1: 50 nM; JVM2/MAVER1: 250 nM) or deferasirox (80 µM). Four hours later, $FeCl_3$ (100 µM) was added to the indicated samples as an exogenous source of iron. After 48 h, cells were washed with PBS, resuspended in PBS with CM-H2DCFDA (1 µM), incubated for 20 min at 37 °C, washed again with PBS, resuspended in RPMI + 10% FBS, and incubated for 2 hours at 37 °C. At the end of the incubation, medium was removed and oxidized CM-H2DCFDA was detected by flow cytometry as FITC fluorescence. RFI: relative fluorescence intensity. Results are the mean of 3-4 independent experiments. **(B)** shows the MFI (Mean Fluorescence Intensity) in control conditions to show that JEKO1 has high levels of ROS in basal conditions. a.u.: arbitrary units. **(C)** Cells were treated as in (A). As a positive control, cells were treated with MitoParaquat (MitoPQ) (5 µM), which selectively increases superoxide production within the mitochondria, for 1 h. Then, cells were incubated with 1 µM MitoSOX at 37 °C for 20 min in HBSS, washed three times with warm HBSS. MitoSOX oxidation signal was acquired directly by flow cytometry as FITC fluorescence. RFI: relative fluorescence intensity. Results are the mean of 3 independent experiments. **(D)** Cells were pre-treated with the ferroptosis inhibitor Ferrostatin-1 (10 µM, 30 min) before treatment with ironomycin (JEKO1: 50 nM; JVM2/MAVER1: 250 nM) or the ferroptosis inducer erastin (4 µM) for 48 h. Lipid peroxidation was analyzed with BODIPY by flow cytometry. Results are the mean of 3-4 independent experiments.

Supplementary Figure S6. (A-C) Cells were grown in RPMI medium supplemented with regular or lipid-free FBS and treated with ironomycin (JEKO1: 50 nM; JVM2/MAVER1: 250 nM) as indicated for 48 h. At the end of the treatment, (A) Annexin V, (B) total ROS and (C) lipid peroxidation were detected by flow cytometry. RFI: relative fluorescence intensity. Results are the mean of 3 independent experiments. Asterisks indicate a significant difference compared to control conditions (untreated + lipids) after applying a Student's t-test for pairs. *: p-value < 0.05; **: p-value < 0.01; ***: p-value < 0.001; ****: p-value < 0.0001; ns: not significant. **(D,E)** Cells were treated with ironomycin (JEKO1: 50 nM; JVM2/MAVER1: 250 nM) for 48 h and (D) the levels of SR-B1 were analyzed by western blot (n = 3) and (E) cells were fixed with 4% formaldehyde for 10 min at RT and incubated with NileRed dye to visualize lipid droplets. DNA was stained with DAPI (blue). Images show one representative out of two independent experiments. Scale bar = 10 µm.

Supplementary Figure S7. Cells were treated with ironomycin (JEKO1: 50 nM; JVM2/MAVER1: 250 nM) or the iron chelator deferasirox (80 µM). Four hours later, $FeCl_3$ (100 µM) was added to the indicated samples as an exogenous source of iron, and cells were incubated for 48 h. Western blot analysis was conducted to study UPR, apoptosis/ferroptosis, and cell cycle/DNA damage factors. Tubulin was used as a loading control. The figure shows one representative out of three independent experiments.

Supplementary Figure S8. (A) Cell lines were treated with increasing concentrations of ibrutinib for 4 days. At day 4, cell viability was assessed using CellTiter-Glo Luminiscent Cell Viability Assay. IC50 for each cell line was calculated using GraphPrism software. Figure shows the

nonlinear regression curve of growth inhibition for all MCLs. Data are based on at least 3 independent experiments. **(B)** Same as Figure 4A. Expression of genes in the BCR signature in each condition is represented. **(C)** Cells were treated as indicated with ironomycin (JEKO1: 50 nM; JVM2/MAVER1: 250 nM) and ibrutinib (JEKO1: 0.5 µM; JVM2: 1.5 µM; MAVER1: 6.25 µM) for 48 h. Cells were collected and whole cell lysates were loaded onto polyacrylamide gels to analyze CARD11 protein levels. Tubulin was used as a loading control. CARD11 signal was normalized to tubulin signal in each condition. Graphs show the mean ± of 3 independent experiments, each one marked with a different color. Lower panels show one representative of 3 independent experiments. Asterisks indicate a significant difference compared to control conditions after applying a Student's t-test for pairs. *: p-value < 0.05; **: p-value < 0.01; ***: p- value < 0.001; ns: not significant. **(D,E)** Cells were treated as in (A) and whole cell lysates were used to analyze the indicated proteins related to BCR signaling by western blot. Tubulin was used as a loading control. Figures show one representative out of 3 independent experiments.

Supplementary Figure S9. Cells were treated as indicated with ironomycin (JEKO1: 50 nM; JVM2/MAVER1: 250 nM) and ibrutinib (JEKO1: 0.5 µM; JVM2: 1.5 µM; MAVER1: 6.25 µM) for 48 h. Cells were collected and whole cell lysates were loaded onto polyacrylamide gels to analyze cell cycle (A), DNA damage (B), and pro-/anti-apoptotic (C,D) related proteins. Tubulin and histone H3 were used as loading controls. Figure shows one representative out of 3 independent experiments.

Supplementary methods:

Gene Expression data analyses and building of the Iron Score

The list of 62 genes involved in the regulation of iron biology was established using previously published data[20,24].

Gene expression microarray data from one cohort (Staudt cohort) of 71 newly-diagnosed MCL patients was used. Lymphochip cDNA microarray gene expression data are publicly available via the online Gene Expression Omnibus (http://www.ncbi.nlm.nih.gov/geo/) under accession number GSE10793[26] . They were performed using Lymphochip cDNA microarray for the cohort of 71 patients. Values used are log2 of 50th percentile normalized mean as reported in GSE10793 dataset.

We used the maximally selected rank test Maxstat R algorithm and Benjamini Hochberg multiple testing correction to define the genes related to iron metabolism that are associated with a significant prognostic value in MCL patients. Multivariate analysis was performed using the Cox proportional hazards model. Survival curves were plotted using the Kaplan-Meier method in the platform Genomicscape[81]. Probe sets with a prognosis value were selected. To gather their prognostic information within one parameter, the Iron Score of MCL was built as the sum of the beta coefficients weighted by ± 1 according to the patient signal above or below the probe set Maxstat value[82,83] .

Culture of primary cells from MCL patients and PBMCs

Primary cells from MCL patients were obtained at the University Hospital of Montpellier after patients written informed consent in accordance with the Declaration of Helsinki and agreement of the Montpellier University Hospital Centre for Biological Resources (DC-2008-417). Cells were cultured in 48-well plates (0.5 million/ml and 0.5 ml/well) in IMDM medium + 10% FBS + CD40ligand (50ng/ml; #2706-CL, R&D) + anti-His (5µg/ml; #MAB050, R&D) in the presence or not of ironomycin (20, 50 and 100 nM) for 4 days. Samples were stained with anti-CD3 (641415, BD Biosciences), anti-CD19 (341113, BD Biosciences), anti-CD20 (655872, BD Biosciences), anti-CD45 (560777, BD Biosciences), and anti-Igκ (F0434, Agilent Technologies) or anti-Igλ (R0437, Agilent Technologies). Tumor cells were detected as Igκ/λ+ CD19+ CD20+ CD45+.

PBMCs were isolated from healthy donors by Ficoll and cultured at $5x10^5$ cells/ml in RPMI 1640 medium + 10% FBS in the presence or not of ironomycin (20, 50 and 100 nM) for 4 days. Samples were stained with anti-CD3, anti-CD19, anti-CD20, anti-CD40 and anti-CD45 to differentiate the different white cells subpopulations: lymphocytes T (CD3+), lymphocytes B (CD19+ CD20+) and monocytes (CD40+).

Cells were acquired with a FACSCanto cytometer and analyzed with Kaluza software.

Cell proliferation and viability

Cells were counted using a Malassez chamber in presence of Trypan blue (15250061, Gibco) dye to distinguish living (-) from dead (+) cells. Proliferation rate was calculated as cell concentration (million/ml) at day 2 divided by the seeding concentration at day 0 (JEKO1/MAVER1: 0.5 million/ml; JVM2: 0.3 million/ml). Cell viability was calculated as the percentage of living cells in each condition compared to the control (100%).

For Ki67 staining, at the end of treatments, cells were fixed and permeabilized with Cytofix/Cytoperm buffer (554722, BD Biosciences) for 10 min at 4°C, washed once with PermWash buffer (554723, BD

Biosciences), and stained with anti-Ki67 antibody or isotypic Alexa-Fluor 647 (558615 and 557714, BD Biosciences). Nuclei were stained in PermWash buffer containing DAPI (2 µg/mL) (Invitrogen, Life Technologies) during 20 min at 4°C. Cells were acquired with a Fortessa cytometer (BD Biosciences) and results were analyzed with Kaluza software.

Cell cycle analysis

For cell cycle analysis, cells were treated as indicated for each experiment. Culture medium was supplemented with 10 µg/ml BrdU (bromodeoxyuridine) 1.5 h before the end of treatments. Cells were collected and processed with "APC BrdU flow kit" (552598, BD Biosciences) according to manufacturer's instructions. Cells were acquired with a Fortessa cytometer (BD Biosciences) and results were analyzed with Kaluza software. BrdU+ cells corresponded to S-phase and BrdU- cells to the G0/G1 or G2/M phases based on their DNA content.

Apoptosis analysis

For apoptosis analysis, cells were treated as indicated for each experiment and stained using "PE Annexin V Apoptosis Detection Kit I" (559763, Becton Dickinson) following manufacturer's protocol. Cells were acquired with a Fortessa cytometer (BD Biosciences) and results were analyzed with Kaluza software.

Quantification of gene expression by qPCR

Cells were treated as indicated for 48 hours. Total RNA was extracted using RNeasy kit from Qiagen (74004) according to the manufacturer's protocol. Purified RNA was quantified using a NanoDrop spectrophotometer and 1 µg was used per sample for retrotranscription using QuantiTect Rev. Transcription Kit (205311, Qiagen) following the manufacturer's instructions. mRNA expression quantification was performed using Taqman probes: Hs00765553_m1 (*CCND1*), Hs00277041_m1 (*CCND2*), Hs01078066_m1 (*RB1*), Hs01565683_g1 (*CDK4*), Hs03003631_g1 (*18S*). Data were normalized to the *18S* housekeeping gene.

Western blot analysis

Cells were directly lysed in Laemmli buffer (400 µl/million cells) and 20 µl/sample were loaded into polyacrylamide gels, and migrated at 40 mA/gel for 1 hour in 1X running buffer (12.5mM Tris-base, 95mM glycine and 0.5% SDS). Proteins were transferred to nitrocellulose membranes (10600002, Amersham Protran, Cytiva®) in 1X transfer buffer (24.8mM Tris-base, 192mM glycine and 20% Ethanol 96%) at 120V for 2 hours. Primary antibodies were incubated in TBS-Tween 20 0.1% (Tris-Buffered Saline, pH 7.4) with 5 % bovine serum albumin (Sigma-Aldrich, A7906) at 4°C overnight.

Antibodies used in this study: Tubulin (12G10 anti-alpha-tubulin, DSHB), phospho-Ser15 (p53) (9284S, Cell Signaling), p53 (9282S, Cell Signaling), Cyclin D1 (55506, Cell Signaling), Cyclin D2 (554201, BD Biosciences), p21 (2946S, Cell Signaling), p27 (3688S, Cell Signaling), phospho-Ser345 (Chk1) (2348S, Cell Signaling), Chk1 (ab40866, Abcam), phospho-Thr68 (Chk2) (2194, Cell Signaling), Chk2 (6334, Cell Signaling), Caspase 3 (9662S, Cell Signaling), Caspase 8 (9746, Cell Signaling), Caspase 9 (9502S, Cell Signaling), PARP (9532S, Cell Signaling), Bcl-xL , Bcl2 (4223S, Cell Signaling), Mcl1 (sc-56152, Santa Cruz), Cytochrome C (ab65311, Abcam), GPX4 (52455, Cell Signaling), BAX (89447, Cell Signaling), BAK (AM03, Calbiochem), BIM (AB17003, Millipore), phospho-Tyr551 (BTK) (18805, Cell Signaling), BTK (8547, Cell Signaling), phospho-Thr308 (Akt) (13038, Cell Signaling), panAkt (4691S, Cell Signaling), phosphor-Ser536 (NF-κB) (3033S, Cell Signaling), NF-κB (3034S, Cell Signaling), phospho-Thr180/Tyr192 (p38) (9211S, Cell Signaling), p38 (9212S, Cell Signaling), γH2AX (05-636, Millipore),

phospho-Ser795(Rb) (9301S, Cell Signaling), Rb (9309S, Cell Signaling), Cdk4 (2906, Cell Signaling), Ferritin (ab75973, Abcam), LC3B (3868S, Cell Signaling), Histone 3 (ab1791, Abcam), SR-B1 (90332T, Cell Signaling).

BH3 profiling

Cells were seeded in 96-well plates at a concentration of 500 000 cells/mL for MAVER and JEKO1 cell lines or 300 000 cells/mL for JVM2, using a robotic liquid handler EPMotion5070. Then, cells were incubated with either DMSO or Ironomycin (JEKO1: 50 nM; JVM2/MAVER1: 250 nM) for 20h, in a 5% CO_2 and 37°C incubator. Next, cells were treated with 3 BH3 mimetics (or vehicle): Venetoclax (Bcl-2 inhibitor), AZD-5991 (Mcl-1 inhibitor), A-1155463 (Bcl-xL inhibitor) at concentrations ranging from 10 nM to 1 µM for 4h. Finally, cells were stained using "APC Annexin V Apoptosis Detection Kit with 7-AAD" (640930, Biolegend) and analyzed by flow cytometry. The gating strategy focuses on live cells to measure Annexin V flipflop as an early apoptotic mark. This assay was paired with a cell viability measurement at 24h, using CellTiterGlo assay (Promega).

Quantification of reactive oxygen species (ROS) and lipid peroxidation

Total reactive oxygen species (ROS) were stained using CM-H2DFDA probe (C6827, Invitrogen). Cells were treated as indicated. As a positive control, cells were treated with H_2O_2 (600 µM) for 1 hour. Then, cells were incubated with 1 µM CM-H2DFDA at 37°C for 20 minutes in PBS, washed once with warm PBS (1500 rpm, 5 min at RT) and incubated during 2 hours at 37°C with RPMI+ 10/20% FBS. Then, cells were washed once with PBS and CM-H2DFDA oxidation signal was acquired directly with the FITC channel of a Fortessa cytometer (BD Biosciences) and results were analyzed with Kaluza software.

Mitochondrial ROS were stained using MitoSOX probe (M36008, Invitrogen). Cells were treated as indicated. As a positive control, cells were treated with MitoParaquat (MitoPQ) (5 µM) for 1 hour. Then, cells were incubated with 1 µM MitoSOX at 37°C for 20 minutes in HBSS, washed three times with warm HBSS (400g, 3 min at RT). MitoSOX oxidation signal was acquired directly with the FITC channel of a Fortessa cytometer (BD Biosciences) and results were analyzed with Kaluza software.

Lipid peroxidation was measured using a BODIPY-C11 581/591 probe (D-3861, Invitrogen). At the end of the treatments, cells were incubated with 5 µM BODIPY-C11 581/591 directly added to the culture medium at 37°C for 30 minutes, then washed three times with PBS and analyzed by flow cytometry. Emission in the PE channel corresponded to non-oxidized lipids and in the FITC channel to peroxidized lipids.

Lipid droplet staining

Cells were deposited on slides using a Cytospin centrifuge (600 rpm, 10 min) and fixed with 4% paraformaldehyde in PBS at RT for 10 minutes. Slides were washed once with PBS and incubated with a solution of PBS + 5 µg/ml of Nile Red (HY-D0718, CliniSciences) at RT in the darkness for 15 min, washed once with PBS and nuclei were stained with DAPI 20 µg/mL diluted in water for 5 minutes at RT protected from light. Slides were washed 3 times with dH_2O, air dried and mounted with Prolong.

RNA sequencing

Cells were treated with ironomycin (JEKO1: 50 nM; JVM2/MAVER1: 250 nM) and/or ibrutinib (JEKO1: 0.5 µM; JVM2: 1.5 µM; MAVER1: 6.25 µM) for 2 days, collected and total RNA was extracted using RNeasy kit from Qiagen (74004) according to the manufacturer's protocol and send to sequencing. The RNA-seq library preparation was done with 150 ng of input RNA (Integragen). Paired-end RNA-seq were performed using HiSeqTM 4000 Illumina machine(Integragen). RNA-seq read pairs were mapped to the reference human genome using the STAR aligner. All statistical analyses were performed with the statistics software R and R packages developed by BioConductor project (available from: https://www.bioconductor.org). The expression level of each gene was summarized and normalized using the DESeq2 R/Bio-conductor package. Differential expression analyses were performed using DESeq2 pipeline. p values were adjusted to control the global FDR across all comparisons with the

default option of the DESeq2 package. Genes were filtered from downstream analysis if they did not have a log2 mean normalized count value of at least 6 in at least one group. Genes were considered differentially expressed if they had an adjusted *p* value < 0.05 and a fold change > 2. Gene Set Expression Analysis (GSEA) was used to identify genes and pathways differentially expressed between groups.

Drug sensitivity tests

Cell lines were seeded in 96-well flat-bottom microtiter plates in the presence of increasing concentrations of ironomycin, deferasirox or ibrutinib, and incubated for 4 days. The number of metabolically active cells in culture, considered as living cells, was determined using Promega's CellTiter-Glo Luminescent Cell Viability Assay (G7573, Promega) according to the manufacturer's protocol, and luminescence measurements were performed using a Centro LB 960 luminometer (Berthold Technologies). The IC50 of each drug was calculated using GraphPad Prism software.

CaspaseGlo Assay

Cells were seeded in 96-well plates and treated in triplicate for each condition. Cells were pre-incubated for 30 min with the pancaspase inhibitor Quinoline-Val-Asp-Difluorophenoxymethylketone (Q-VD-Oph, 20 µM) before treatment with ironomycin (JEKO1: 50 nM, JVM2/MAVER1: 250 nM) or bendamustin (20 µM) for 48 hours. At the end of the treatments, the activities of caspases 3/7, caspase 8 and caspase 9 were analyzed using the corresponding CaspaseGlo Assays (CaspaseGlo® 3/7 assay (G8091), Caspase Glo® 8 assay (G8200) and CaspaseGlo® 9 assay (G8210), all from Promega). The assay was performed according to the manufacturer's protocol and read with a Centro LB 960 luminometer (Berthold Technologies). The results were normalized to control conditions in each case.

Iron supplementation

Cells were seeded in 24-well plates (1 ml/well) at their regular seeding concentration (see "Mantle Cell Lymphoma cell lines culture") and directly treated with ironomycin (JEKO1: 50 nM, JVM2/MAVER1: 250 nM) or the iron chelator deferasirox (80 µM). Four hours later, $FeCl_3$ (100 µM) was added to the indicated samples as an exogenous source of iron, and cells were incubated for 2 days. At the end of the treatments, cells were collected, stained using "PE Annexin V Apoptosis Detection Kit I" (559763, Becton Dickinson) following manufacturer's protocol. Cells were acquired with a Fortessa cytometer (BD Biosciences) to detect Annexin V+ cells and results were analyzed with Kaluza software.

LC3BII Immunofluorescence

All steps were carried out at room temperature (RT). Cells were deposited on slides using a Cytospin centrifuge (600 rpm, 10 min) and fixed with 4% paraformaldehyde for 10 minutes, permeabilized with 0.5% Triton in phosphate-buffered saline (PBS) for 10 minutes and saturated with 5% bovine milk in PBS for 30 minutes. The rabbit anti-LC3B antibody (3868S, Cell Signaling) was diluted 1:300 in 5% bovine milk in PBS, and deposited on the slides for 1.5 hour. Slides were washed twice (5 min) with PBS-Tween 0.01% and secondary antibody anti-rabbit Alexa Fluor 488 (A11008, diluted 1/500 in 5% bovine milk in PBS) was added for 45 min at room temperature. Slides were washed three times (5 min) in PBS-Tween 0.01% and incubated 5 min with 20 µg/ml DAPI in ddH2O. Then the slides were washed with water and mounted on glass slides using ProLongTM Gold antifade reagent (36930, Life technologies, Thermofisher). Images and fluorescence were captured with a ZEISS Axio Imager Z3 microscope (× 40 objective), analyzed with Zen software and Omero software.

CD71 quantification by flow cytometry

Cells were treated as indicated for 48 hours, collected, washed once with cold PBS (1500 rpm, 5 min, 4°C) and incubated with 5 µl of anti-CD71 (ref) antibody for 20 min at 4°C. Then, cells were washed once with cold PBS, resuspended in PBS and acquired with Fortessa cytometer. Data were analyzed with Kaluza software.

Intracellular ATP quantification

Cells were seeded in 6-well plates (5 ml/well) and treated with increasing concentrations of ironomycin as indicated for 48 hours. At the end of the treatment, cells were counted, centrifuged and resuspended in warm RPMI + 10/20% FBS at $5x10^5$ cells/ml and 50 µl were deposited in an opaque 96-well plate. Then, 50 µl/well of CTG were added, incubated for 10 min at RT protected from light and luminescence was read with a Centro LB 960 luminometer (Berthold Technologies). Results were normalized to control conditions for each cell line and expressed as percentage.

Seahorse assay

JEKO1, JVM2 and MAVER1 cell lines were treated with Ironomycin for 2 days prior to Seahorse assay. Cellular oxygen consumption rate (OCR) was measured using an XFe96 Extracellular Flux Analyser (Agilent Seahorse, Billerica, MA, USA) and the Mito Stress Test Kit, according to the manufacturer's protocol. Briefly, seahorse 96-well plate was coated with Cell-Tak mixture (77,8µl of Cell-Tak solution in 5ml of bicarbonate sodium pH8) the previous day. On the day of the experiment: XFe/XF96 sensor cartridge was moved from H2O into pre-warmed XF calibrant for 45 to 60min prior to loading the injection port with the Mito-stress assay compounds. The compounds were prepared in Seahorse XF RPMI Media and used at the following concentration: Oligomycin (1,5µM), FCCP (1µM), Rot/AA (0,5µM). The cells were counted and resuspended in Seahorse XF RPMI Media (supplemented with pyruvate (100mM final), glutamine (200mM) and 10mM glucose). Cells (0,2M/well) were then attached to Cell-Tak-coated plate and incubated at 37°C for 45min (without CO2). Plate was then run on the bioanalyzer Seahorse XFe96 and analysis performed using the Seahorse Analytics portal.
