# Supplementary material for: Ironomycin induces mantle cell lymphoma cell death by targeting iron metabolism addiction
Source: Theranostics. 2025 Feb 3;15(7):2834–51. doi: 10.7150/thno.101821 (PMC11898298; doi:10.7150/thno.101821)
Supplement: Supplementary file 3 — Supplementary tables 2-3. [file thnov15p2834s3.pdf]

**GENETIC ALTERATION**	**JEKO1**	**JVM2**	**MAVER1**	**GRANTA519**	**MINO**	**REC1**
**t(11;14)**	+	+	+	+	+	+
**Expression of Cyclin D1**	+	weak	+	+	+	+
**Expression of Cyclin D2**	-	+	-	-	+	-
SOX11	+	-		+	+	+
**cMYC (rearrangement 8q24)**	+	+	+	+/-	+/-	+/-
RB1	+			+		+
TP53	del/mut	wt/wt	mut/del	wt/wt	del/mut	wt/wt
ATM	amplified	wt	del/nd	wt	mutated	del/nd
***CDKN2A* (p16)**	+	+	del	del	+	+
***CDKN1A* (p21)**	+	+	mut	+	+	-
***CDKN1B* (p27)**	+	-	nd	+	+	+
**SR-B1**	-	++	+			
BCL2	+	+	+	+		

Supplementary table S2: Characteristics of the different MCL cell lines.

CELLULAR RESPONSE	JEKO1	JVM2	MAVER1
**Ironomycin IC50 (nM)**	12.8	112.6	50.4
**Total ROS production (tROS)**	-	++	+
**Mitochondrial ROS production (mROS)**	+	+	-
**Cell death**	Apoptosis ++<br>Ferroptosis ++	Apoptosis ++<br>Ferroptosis +	Apoptosis +<br>Ferroptosis ++
**Pro- / anti-apoptotic proteins expression**	↓	↑	↑
**Oncogene degradation**	CCND1 ↓<br>p27 ↓	CCND1 ↓<br>CCND2 ↓<br>p53 ↓	CCND1 ↓<br>p53 ↑<br>p21 ↑<br>p27 ↑
**BH3 mimetic priming**	Bcl2i<br>Mcl1i<br>Bcl-xLi	Bcl2i	Bcl2i
**UPR activation**	IRE1α ++<br>BiP ++<br>CHOP +	IRE1α +<br>peIF2α ++<br>CCPG1 ++	IRE1α ++<br>XBP1s +<br>BiP ++<br>ATF4 ++<br>CCPG +
**GPX4**	↓	↑	↑
**Cell cycle**	Sub-G1<br>G2/M	Sub-G1	G0/G1
**FeCl3 supplementation**	↑ tROS<br>↓ mROS	↑ tROS<br>↑ mROS	= tROS<br>↓ mROS
**Lipid depletion**	↑ cell death<br>↑ lipid peroxydation<br>↓ tROS	↑ cell death<br>= lipid peroxydation<br>↑ tROS	↑ cell death<br>↓ lipid peroxydation<br>↓ tROS

Supplementary table S3: The different cellular effects of ironomycin in the three MCL cell lines studied are summarized in the table.
